# POPUP: an observational digital study reporting general population norms for the EQ-5D-5-L and HUI-3 in 8 countries

**DOI:** 10.1186/s13690-025-01642-z

**Published:** 2025-07-11

**Authors:** Sarah Dewilde, Nafthali Hananja Tollenaar, Glenn Phillips, Sandra Paci, Mathieu F. Janssen

**Affiliations:** 1 Services in Health Economics (SHE), Boulevard Lambermont 418, Brussels, 1030 Belgium; 2 argenx BV, Ghent, Belgium; 3 Section Medical Psychology and Psychotherapy, Department of Psychiatry, Erasmus MC, Rotterdam, The Netherlands

**Keywords:** EQ-5D-5L, Health utility index, HUI-3, Health-related quality of life, HRQoL, Population norms, General population

## Abstract

**Background:**

This study aimed to estimate population norms in the US, Canada, UK, Italy, Spain, Germany, The Netherlands, and Belgium for the EQ-5D-5-L with six bolt-on dimensions (vision, breathing, tiredness, sleep, social relationships, self-confidence), and for the Health Utilities Index-Mark 3 (HUI-3).

**Methods:**

A digital study was conducted among 9,000 general population participants, representative of age, sex, education, and region within each country. Data collection included demographics, health conditions, EQ-5D-5-L and bolt-ons, and the HUI-3. National population norms were calculated for each dimension and for utility values. Testing for differences between subgroups was performed with a Generalized Linear Model.

**Results:**

The proportion of respondents reporting severe-to-extreme problems at dimension level was highest on the EQ-5D-5-L dimensions pain/discomfort (5.5%) and anxiety/depression (5.6%), and on the HUI-3 dimensions pain (5.7%), emotion (5.4%), and cognition (4.1%). Severe-to-extreme problems on the EQ-5D-5-L bolt-on dimensions were social relationships (8.0%), sleep (7.6%), tiredness (7.4%), self-confidence (5.1%), vision (3.7%), and breathing (2.0%). Mean EQ-5D-5-L utility values for all countries combined displayed a U-shape by age and ranged between 0.819 and 0.871, whereas HUI-3 utility values ranged between 0.717 and 0.768 without a clear pattern. The impact of age by sex on EQ-5D-5-L utility values was country-specific. HUI-3 utilities did not show a linear trend by age, and no difference was found by sex. Italy had the highest mean EQ-5D-5-L utility values, while the Netherlands and Spain had the highest values according to the HUI-3. The lowest utility values were observed in the UK, for both instruments. Utility values differed significantly by education, employment, place of residence, needing a caregiver, being on sick leave and having health conditions such as dementia, MS, depression, rheumatoid arthritis, systemic lupus erythematosus and heart failure.

**Conclusions:**

Important differences in reporting problems and in utility values were found between countries and subgroups, highlighting the need for country-specific population norms.

**Supplementary Information:**

The online version contains supplementary material available at 10.1186/s13690-025-01642-z.

**Table:** 

Text box 1: Contributions to the literature
• Population norms show how the general population rates their health-related quality of life, and generic instruments like the EQ-5D-5-L and HUI-3 have been used to measure it in patients and in the general population.
• Existing EQ-5D-5-L population norms are sometimes outdated or based on small samples. HUI-3 population norms exist only for the US and Canada.
• This study gathered data from 9,000 people across eight countries and found notable differences by country, age, and sex– highlighting the need for country-specific reference values.
• Population norms can help understanding the burden of health conditions and evaluate treatment effectiveness.

## Background

Health-related quality of life (HRQoL) has become an increasingly important outcome in health care decision-making, especially when assessing the influence that long-term illness or chronic conditions may have on people’s lives and how treatments can impact HRQoL [1]. *Multidimensionality and subjectivity* have been identified as two fundamental aspects of HRQoL. The former pertains to the comprehensive nature of HRQoL, which encompasses various dimensions of health and well-being (including physical, functional, emotional, and social well-being) associated with activities of daily life such as work, leisure, household management, and interpersonal relationships. The latter refers to the notion that HRQoL is best measured from the subject’s perspective [2].

Many generic and disease-specific instruments have been developed to measure HRQoL and are usually optimized to capture its *multidimensionality* aspects [3]. Among the most commonly used generic instruments are the EQ-5D-5-L [4] and the HUI-3 [5], both intentionally designed to be brief and simple HRQoL measures covering a set of dimensions that are arguably the most relevant to overall health. These instruments describe an individual’s health state and assign a corresponding utility value reflecting their overall health status. Utility values are derived from population preferences for specific health states within a given country (i.e., country-specific value sets) and can be used to calculate quality-adjusted life years (QALYs) [6], the recommended health outcome for economic evaluations of healthcare interventions in many countries, including Canada, the United Kingdom (UK), Italy, Spain, Germany, the Netherlands, and Belgium, but not universally (e.g., not formally in the United States [US]). By applying country-specific value sets to large, representative population samples, population norms can be established, i.e., benchmarks that reflect the average HRQoL in the general population [6].

Population norms can be used to compare the HRQoL of specific (patient) populations to the HRQoL of members of the general population, enabling the quantification of disease burden in, for example, rare diseases [7]. Additionally, they facilitate the comparison of efficacy outcomes in interventional trials [8]. EQ-5D population norms have been published for many countries [8–10], but data is at times outdated or based on the EQ-5D-3 L– an earlier and less extended version of the EQ-5D-5-L. Contrary to the EQ-5D-5-L, HUI-3 population norms have only been published for the US [11] and Canada [12] and not for any European country.

Since generic HRQoL measures are designed to be broadly applicable and complementary to condition-specific measures, they may lack content validity and thus be less sensitive in capturing the specific dimensions of a particular health condition [13]. Examples of dimensions known to significantly impact HRQoL include sensory impairments (vision, hearing, smell, taste) [14], specific aspects of physical functioning (breathing, sleeping, fatigue/energy), mental health-related dimensions (emotions, self-confidence) or a person’s social role (social relationships) [15]. So far, approximately 30 different so called “bolt-on” dimensions have been proposed for the adult EQ-5D instruments to improve their content validity and sensitivity to change in specific populations or contexts [16]. However, population norms are also lacking for these bolt-on dimensions [17]. Knowledge of the distribution of bolt-on dimensions in the general population can help researchers and policymakers identify and interpret QALY differences between interventions targeting general populations, while also improving the sensitivity of the EQ-5D in specific (patient) populations [13].

The objective of this study, referred to as the Population Norms Study (POPUP), was therefore to derive population norms for both the EQ-5D-5-L and HUI-3 HRQoL measures among representative samples of the general population from eight countries; and to report population norms for extensions of the EQ-5D-5-L, namely for six bolt-ons covering vision, breathing, tiredness, sleep, self-confidence, and social relationships. These bolt-ons were selected for their relevance to general HRQoL, broad applicability across populations, and their potential to serve as baseline norms for comparing health outcomes in populations with specific health conditions [18].

## Methods

The POPUP study, is a multinational digital survey designed to derive HRQoL norms from a general population sample of 9,000 participants across eight countries (Belgium, Canada, Germany, Italy, the Netherlands, Spain, the United Kingdom [UK], and the United States [US]) [19]. Samples sizes were not based on formal calculation but driven by the aim of representativity for the general population. However, a sample size of 1,000 with a margin error (ME) of 3.2% (ME% = 100 / √N) was considered acceptable for a survey among the general population [20]. A larger sample of 2,000 was collected in the US to account for its substantially larger and more diverse population, ensuring more reliable and representative estimates for national-level analyses. In all eight countries, samples were drawn from panels of willing survey participants using stepped random sampling to ensure diversity and representation across key demographic variables (age, sex, education, and region) [21, 22]. The sampling process was carried out in multiple stages, beginning with broad panels, and progressively narrowing down to smaller, selected groups based on recruitment progress. This multi-stage approach helped ensure that individuals from various regions and socioeconomic backgrounds were included. After the sampling, propensity weights were applied to adjust the sample to better reflect the general population, compensating for any imbalances introduced during the multi-stage sampling process [21, 22]. The representative panels, operated by an international market research company [23], contain a large number of participants, regularly updated with new entries while filtering out individuals flagged for survey speeding or failing trap questions and quality checks. Only panel members could participate in the survey. No online sampling methods, such as river sampling, routing, or real-time web sampling, were used. Respondents were recruited in the panel from various sources (TV, radio, newspapers). Potential participants were invited via e-mail and offered points for completing the online survey [23], which could later be converted to a selection of gifts. The survey questionnaires were programmed and hosted on the LimeSurvey web portal by a research company with expertise in data collection [24]. To optimize the quality and efficiency, we ran a pilot study (of which the data were not analyzed) pre-testing the survey with 10% of the sample in each country. Additionally, we limited the responses from the entire possible range to the plausible range of responses for several questions. We cleaned the data by removing participants who rushed through the survey (< 300 s) and checked for validity and internal consistency to ensure high data quality and completeness.

This study reported population norms based on data collected at baseline in January-March 2021. Demographic characteristics such as age, sex, level of education, country of residence, living situation, employment situation, and presence of health conditions were collected, in addition to the EQ-5D-5-L plus the six bolt-ons, and the HUI-3. No names or other personal identifiers were recorded. Permissions were obtained for the use of the instruments from the EuroQol Group and from Health Utilities Inc. Ethical approval was obtained in all eight countries. The study was authorized by Veritas IRB in Canada (reference number 2021-2434-5740-1), the ethics committee at Ghent University Hospital in Belgium (reference number BC-07857) and Salus IRB reviewed and approved the other countries (reference number PN8450).

### EQ-5D-5-L and bolt-ons

The EQ-5D-5-L consists of a descriptive system assessing general health on 5 dimensions: mobility (MO), self-care (SC), usual activities (UA), pain/discomfort (PD) and anxiety/depression (AD). Each dimension is described in 1 to 5 severity levels ranging from “having no problems”, “having slight problems”, “having moderate problems”, “having severe problems” to “having extreme problems / being unable” to perform the action on that dimension on the day of completion [4]. Responses on the five core dimensions can be combined into a utility value, which summarizes overall HRQoL. This value is anchored between 1 (representing full health), 0 (dead), with negative values indicating health states perceived to be worse than death. A set of utility values, known as a country-specific tariff represents the general population’s preferences for a given health state as described by the EQ-5D-5-L. Furthermore, the visual analogue scale (EQ VAS) is a thermometer-like vertical scale ranging from 0 (worst imaginable health) to 100 (best imaginable health) on which respondents rate their overall health on the day of completion. Country-specific language versions approved by the EuroQoL Group were used. Several previously developed and published bolt-ons were added to the standard EQ-5D-5-L dimensions including vision [25], breathing [26] sleep [27], tiredness [25], social relationships [28], and self-confidence [28] with the same five response levels as the main EQ-5D descriptive system [18].

### Health utilities index 3 (HUI-3)

The HUI-3 is a generic, preference-based, comprehensive system for measuring the current health status of participants and their HRQoL [5]. The HUI-3 classification system distinguishes eight dimensions of health: vision, hearing, speech, ambulation, dexterity, emotion, cognition, and pain. Each level is described with 5 or 6 levels of ability/disability [5]. HUI-3 responses can also be summarized into a utility value, ranging from − 0.36 (worst heath state) to 1 (full health) with 0 as an anchor representing dead. The HUI-3 utilities have good correlation with EQ-5D utilities, but are not interchangeable [29].

### Statistical analysis

Firstly, demographics characteristics (age, sex, level of education, and living situation) and the prevalence of self-reported health conditions were summarized for each country using descriptive statistics, and compared to national data if available. While we used three age groups (18–34, 35–54, 55+) to align our sample with national statistics for the purpose of propensity weighting, we stratified utility values by age groups with narrower categories (18–24, 25–34, 35–44 etc.) to present a more detailed insight on the evolution of HRQoL across different life stages. Level of education was assessed using a single question with three standardized response options: primary, secondary, and higher education. These categories were applied consistently across all countries, without adjustment for differences in national education systems.

We estimated reference distributions across levels of ability and disability of each countries’ general population, by calculating the proportion of responders per level on each dimension of the EQ-5D-5-L (including bolt-ons and EQ VAS scores) and HUI-3 instruments. Additionally, EQ-5D-5-L utility values were calculated for each country by applying country-specific tariffs to the profile data [30–37]. Mean utility values for the entire sample were generated by taking an arithmetic average of the utility values for each country. HUI-3 utility values were calculated using the Canadian value set for all countries, the only available value set [38]. Utility values and EQ VAS scores are presented using the mean, standard deviation (SD), and the inter-quartile range (Q1-Q3). Spearman’s rank correlation was used to report the association between EQ-5D-5-L utility values and EQ VAS scores. A moderate correlation (*r* = 0.40–0.59) [39] is expected, as EQ VAS reflects overall self-rated health (*subjectivity*), while utility values are based on specific health dimensions (*multidimensionality*).

We used Generalized Linear Models (GLM) to model and test for differences in utility values for subgroups defined by age groups, sex, and country (together in one multivariable regression model), and to estimate the utility value of subgroups defined by place of residence, education level, employment, and the presence of comorbidities (in univariable regression models). Given that EQ-5D-5-L utility values often exhibit bimodal and skewed distributions, we tested alternative GLM specifications using different combinations of conditional distributions (normal and gamma) and link functions (identity and log). Model fit was assessed using the log-likelihood, Akaike Information Criterion (AIC), and Bayesian Information Criterion (BIC). Based on these metrics, the GLM with a normal distribution and identity link function– equivalent to an ordinary least squares (OLS) model– provided the best fit to the data and was therefore selected as the final model. We used SAS version 9.4 to perform all statistical analyses.

## Results

### Study participants

A total of 9,000 members of the general population from the eight countries participated in the POPUP study, with 1,000 respondents per country, except for the US, where there was 2,000 respondents to account for its large population (Table 1). A large proportion of individuals (35.3% in the UK to 39.1% in Germany) fell within the age range of 35 to 54 years. The composition of the national statistics, and hence our sample, demonstrated a slight preponderance of females (50.4% in Belgium to 52.3% in Italy) compared to males. The proportion of higher educated respondents ranged from 26.3% in Germany to 54.3% in Canada. In all countries, the majority of participants live at home without help from a caregiver (58.4 in the UK to 91.0% in the Netherlands). EQ VAS scores show that the general population in POPUP rated their own health on average at 76.3 on a scale of 100, ranging from 72.3 in the UK to 79.0 in the US. Anxiety (14.7%) and depression (11.5%) were the most observed health conditions in our sample, followed by thyroid problems/disorder (9.7%), diabetes (9.2%), and respiratory disease (8.8%) (See Online Supplemental Material, Table S1).

**Table 1:** Patient characteistics

	Belgium		Canada		Germany		Italy		Netherlands		Spain		UK		US	
Proportion of responders	POPUP	National statistics	POPUP	National statistics	POPUP	National statistics	POPUP	National statistics	POPUP	National statistics	POPUP	National statistics	POPUP	National statistics	POPUP	National statistics
	*N* = 1000	*N* = 9.3*	*N* = 1000	*N* = 31.2*	*N* = 1000	*N* = 69.5*	*N* = 1000	*N* = 50.0*	*N* = 1000	*N* = 14.3*	*N* = 1000	*N* = 39.5*	*N* = 1000	*N* = 53.4*	*N* = 2000	*N* = 264.1*
**Age**																
18–34	28.3%	28.3%	29.4%	29.4%	24.1%	24.1%	22.9%	22.9%	26.3%	26.3%	27.0%	27.1%	28.9%	28.9%	30.6%	30.6%
35–54	35.7%	35.7%	39.1%	39.1%	36.2%	36.2%	37.0%	37.0%	37.4%	37.4%	38.4%	38.4%	35.3%	35.3%	36.7%	36.7%
55 +	36.0%	36.0%	31.6%	31.6%	39.7%	39.7%	40.2%	40.2%	36.4%	36.4%	34.6%	34.5%	35.8%	35.8%	32.7%	32.7%
**Sex**																
Female	50.4%	50.4%	50.5%	50.5%	51.7%	51.7%	52.3%	52.3%	51.0%	51.0%	51.2%	51.1%	51.5%	51.5%	51.4%	51.4%
Male	49.7%	49.7%	49.6%	49.6%	48.3%	48.3%	47.7%	47.7%	49.1%	49.1%	48.9%	48.9%	48.6%	48.6%	48.6%	48.6%
**Education**																
Primary education	3.4%	59.9%	2.0%	46.0%	6.4%	70.1%	6.7%	51.0%	1.7%	66.9%	11.2%	73.8%	1.8%	69.0%	4.4%	64.7%
Secondary education	56.5%		43.7%		67.4%		42.9%		60.0%		61.9%		62.4%		55.6%	
Higher education	40.1%	40.1%	54.3%	54.0%	**26.3%**	**29.9%**	50.4%	49.0%	**38.3%**	**33.1%**	27.0%	26.2%	**35.8%**	**31.0%**	**39.9%**	**35.3%**
**Living situation**																
At home without	76.2%		62.6%		72.9%		68.1%		91.0%		79.4%		58.4%		62.4%	
help from a																
caregiver																
At home with	0.9%		2.0%		1.7%		1.3%		4.3%		2.0%		3.5%		6.3%	
help from a																
caregiver																
With a family	22.6%		35.3%		25.2%		30.0%		4.6%		18.5%		37.7%		30.6%	
member																
In a nursing	0.3%		0.2%		0.1%		0.5%		0.0%		0.1%		0.1%		0.6%	
home																
In a long-term care	0.1%		0.1%		0.0%		0.0%		0.0%		0.0%		0.3%		0.1%	
rehabilitation facility																
**EQ VAS**, Mean	76.2	77.6	73.9	n/a	76.8	77.3	76.1	77.1	76.9	82.0	76.9	75.0	72.3	82.8	79.0	80.0

*Adult population; number in millions of people. Differences between observed characteristics and national statistics are highlighted in bold. The prevalence of health conditions in the POPUP sample is available in the Online Supplemental Material, Table S1

### EQ-5D-5-L dimensions responses

Distribution of responses for all EQ-5D-5-L dimensions are displayed per country in Table 2. The proportion of participants reporting “no problems” on all five EQ-5D-5-L dimensions, was 32.7% across the entire sample, highest in the US (40.0%), followed by the Netherlands (37.4%) and lowest in Germany (26.2%) and Canada (26.6%). Results by dimension show that pain/discomfort and anxiety/depression were the dimensions most frequently associated with the reporting of severe or extreme problems (5.5% and 5.6% overall). Severe to extreme problems in any dimension (level 4–5) were most frequently observed in the UK (13.9%) and Canada (13.8%), and the least in Italy (6.6%). Of note, respondents from English-speaking countries indicated twice as often that they had extreme problems in any dimension compared to continental European countries. For example, about 3% of respondents from US, UK and Canada reported to experience severe anxiety/depression, which is about three times higher than the continental European countries.

**Table 2:** Distribution of the respondents across the 5 dimensions of the EQ-5D-5L

Dimensions	Belgium	Canada	Germany	Italy	Netherlands	Spain	UK	US	All countries
Sample size	*N* = 1000	*N* = 1000	*N* = 1000	*N* = 1000	*N* = 1000	*N* = 1000	*N* = 1000	*N* = 2000	*N* = 9000
**Mobility**									
No problems	76.7%	75.3%	68.8%	80.0%	74.3%	82.5%	74.1%	75.7%	75.9%
Mild problems	14.8%	14.5%	17.4%	13.2%	16.7%	12.2%	15.4%	13.8%	14.6%
Moderate problems	6.7%	6.1%	9.6%	4.7%	5.9%	3.8%	6.3%	6.7%	6.3%
Severe problems	1.6%	2.9%	4.2%	1.9%	2.5%	0.9%	3.6%	2.7%	2.6%
Extreme problems	0.3%	1.2%	0.1%	0.2%	0.7%	0.7%	0.7%	1.2%	0.7%
**Self-Care**									
No problems	93.3%	89.7%	90.7%	89.9%	92.3%	92.5%	86.8%	88.0%	90.1%
Mild problems	4.9%	6.1%	6.5%	7.3%	5.5%	5.6%	6.8%	6.6%	6.2%
Moderate problems	1.1%	2.9%	2.1%	1.7%	1.7%	0.7%	5.0%	3.0%	2.4%
Severe problems	0.4%	0.5%	0.1%	0.6%	0.6%	0.8%	0.9%	1.7%	0.8%
Extreme problems	0.3%	0.9%	0.5%	0.5%	0.0%	0.4%	0.5%	0.7%	0.5%
**Usual Activities**									
No problems	77.4%	74.0%	72.5%	82.7%	72.3%	83.1%	72.4%	77.4%	76.6%
Mild problems	14.7%	18.1%	16.3%	12.1%	16.4%	10.1%	15.9%	13.0%	14.4%
Moderate problems	5.4%	5.6%	8.3%	4.2%	8.4%	5.1%	6.4%	6.4%	6.2%
Severe problems	2.1%	1.6%	2.5%	0.6%	2.2%	1.5%	3.7%	2.6%	2.2%
Extreme problems	0.4%	0.8%	0.4%	0.4%	0.6%	0.2%	1.5%	0.6%	0.6%
**Pain / Discomfort**									
No problems	42.4%	43.6%	36.0%	46.4%	52.5%	52.4%	49.4%	52.3%	47.5%
Mild problems	35.4%	36.8%	40.0%	37.7%	31.2%	32.3%	31.2%	27.8%	33.3%
Moderate problems	17.1%	14.7%	17.4%	13.8%	11.0%	9.9%	12.6%	13.4%	13.7%
Severe problems	4.7%	4.1%	5.7%	2.0%	5.1%	4.5%	4.9%	4.4%	4.4%
Extreme problems	0.4%	0.9%	1.0%	0.1%	0.2%	1.0%	1.9%	2.1%	1.1%
**Anxiety / Depression**									
No problems	59.0%	47.9%	58.0%	55.4%	67.3%	53.3%	51.3%	62.5%	57.5%
Mild problems	25.2%	30.3%	24.7%	29.4%	20.1%	32.0%	25.8%	20.6%	25.4%
Moderate problems	11.3%	14.2%	10.8%	11.9%	8.4%	10.1%	15.1%	11.0%	11.5%
Severe problems	3.3%	5.0%	5.5%	2.6%	3.6%	3.4%	4.8%	2.8%	3.8%
Extreme problems	1.3%	2.7%	1.1%	0.7%	0.7%	1.2%	3.0%	3.1%	1.9%
**Profiles**									
Number of different profiles	160	184	174	147	172	115	174	258	592
% no problems in every dimension	30.7%	26.6%	26.2%	30.6%	37.4%	32.8%	30.3%	40.0%	32.7%
% severe or extreme in at least one	10.0%	13.8%	13.5%	6.6%	10.8%	9.7%	13.9%	11.8%	11.3%
dimension									
% negative utility values	0.5%	0.6%	0.4%	0.5%	0.6%	0.5%	2.3%	2.0%	1.0%

### EQ-5D-5-L bolt-ons responses

Table 3 displays the distribution of respondents across the levels of the EQ-5D-5-L bolt-ons. In POPUP, 3.7% of respondents reported having severe-to-extreme problems with vision (this was highest in Belgium, and lowest in the UK), whereas 41.9% had no problems and 54.4% had mild to moderate problems. Severe-to-extreme problems with breathing were reported by 2.0% of the POPUP sample, with the highest prevalence observed in the US and the lowest in Spain and Italy. The majority (77.3%) of participants reported no breathing problems, and 20.7% reported mild to moderate problems. Overall, 7.6% reported severe-to-extreme problems on the sleep dimension (this was the highest in Germany and the lowest in Canada and Italy), whereas 42.3% had no problems and 50.2% mild to moderate problems. Severe-to-extreme problems with tiredness were reported by 7.4% of the POPUP sample (highest in Germany, and four times lower in Italy). Half of the respondents did not experience any problems with tiredness and 44.6% reported mild to moderate problems. Two-thirds of the respondents in POPUP did not have any problems with self-confidence, whereas 28.1% had mild to moderate problems and 5.1% severe-to-extreme problems (this was the lowest in Italy, and markedly higher in English-speaking countries and Germany). Lastly, severe-to-extreme problems with social relationships were present in 8.0% of the POPUP sample (this was the highest in the UK and the lowest in Italy and the Netherlands), whereas 53.9% had no problems and 38.2% mild to moderate problems.

**Table 3:** Distribution of the respondents across the 6 bolt-on questions to the EQ-5D-5L

Dimensions	Belgium	Canada	Germany	Italy	Netherlands	Spain	UK	US	All countries
Sample size	*N* = 1000	*N* = 1000	*N* = 1000	*N* = 1000	*N* = 1000	*N* = 1000	*N* = 1000	*N* = 2000	*N* = 9000
**Vision**									
No problems	33.4%	46.2%	39.6%	22.3%	44.7%	26.2%	54.5%	55.1%	41.9%
Mild problems	39.1%	31.3%	36.7%	40.5%	34.2%	45.1%	29.8%	28.3%	34.8%
Moderate problems	22.1%	18.6%	19.5%	34.4%	16.7%	25.9%	13.3%	13.1%	19.6%
Severe problems	5.3%	3.4%	3.7%	2.9%	3.6%	2.4%	2.2%	2.5%	3.2%
Extreme problems	0.1%	0.6%	0.6%	0.0%	0.8%	0.5%	0.2%	1.0%	0.5%
**Breathing**									
No problems	76.5%	78.7%	73.2%	79.2%	78.2%	78.0%	76.7%	77.8%	77.3%
Mild problems	16.5%	13.7%	18.9%	15.4%	14.6%	16.1%	16.8%	13.7%	15.5%
Moderate problems	5.8%	5.9%	6.1%	4.5%	5.4%	5.1%	5.1%	4.6%	5.2%
Severe problems	0.9%	1.0%	1.3%	0.9%	1.4%	0.5%	1.3%	2.2%	1.3%
Extreme problems	0.3%	0.8%	0.6%	0.0%	0.4%	0.2%	0.0%	1.8%	0.7%
**Sleep**									
No problems	39.8%	36.6%	30.7%	42.5%	49.3%	44.5%	35.1%	50.9%	42.3%
Mild problems	37.8%	38.7%	41.7%	37.7%	31.1%	38.2%	36.2%	27.6%	35.2%
Moderate problems	15.3%	18.5%	18.4%	13.6%	11.7%	10.5%	20.6%	13.2%	15.0%
Severe problems	5.5%	4.1%	8.0%	5.2%	6.4%	5.4%	5.0%	4.9%	5.5%
Extreme problems	1.7%	2.1%	1.3%	1.0%	1.5%	1.4%	3.2%	3.4%	2.1%
**Tiredness**									
No problems	45.9%	43.7%	41.9%	54.9%	53.9%	47.6%	39.8%	52.5%	48.1%
Mild problems	31.5%	32.3%	31.3%	31.5%	28.7%	30.7%	32.3%	24.8%	29.8%
Moderate problems	15.5%	16.8%	15.2%	10.9%	12.4%	17.3%	19.7%	12.7%	14.8%
Severe problems	6.0%	5.4%	8.9%	2.3%	4.0%	3.9%	5.5%	5.9%	5.3%
Extreme problems	1.1%	1.8%	2.6%	0.3%	1.1%	0.5%	2.7%	4.2%	2.1%
**Self-confidence**									
No problems	66.2%	58.2%	63.3%	72.7%	72.7%	70.6%	61.2%	68.3%	66.8%
Mild problems	21.6%	24.3%	18.8%	17.3%	16.7%	16.1%	22.1%	15.6%	18.7%
Moderate problems	8.6%	11.0%	11.7%	7.5%	7.3%	8.9%	10.5%	9.7%	9.4%
Severe problems	2.9%	4.6%	5.0%	2.2%	2.4%	3.5%	4.1%	3.3%	3.5%
Extreme problems	0.8%	2.0%	1.3%	0.3%	0.9%	0.9%	2.0%	3.2%	1.6%
**Social Relationships**									
No problems	48.4%	49.5%	51.3%	61.9%	59.3%	53.4%	39.7%	60.7%	53.9%
Mild problems	29.3%	27.1%	27.2%	21.1%	25.2%	25.5%	32.3%	21.7%	25.7%
Moderate problems	14.6%	14.3%	14.0%	11.2%	9.8%	12.8%	17.1%	9.4%	12.5%
Severe problems	5.9%	5.8%	5.5%	4.1%	4.6%	6.0%	7.3%	4.2%	5.3%
Extreme problems	1.9%	3.3%	1.9%	1.7%	1.2%	2.3%	3.6%	4.0%	2.7%

### EQ-5D-5-L utility values

**Fig. 1:**
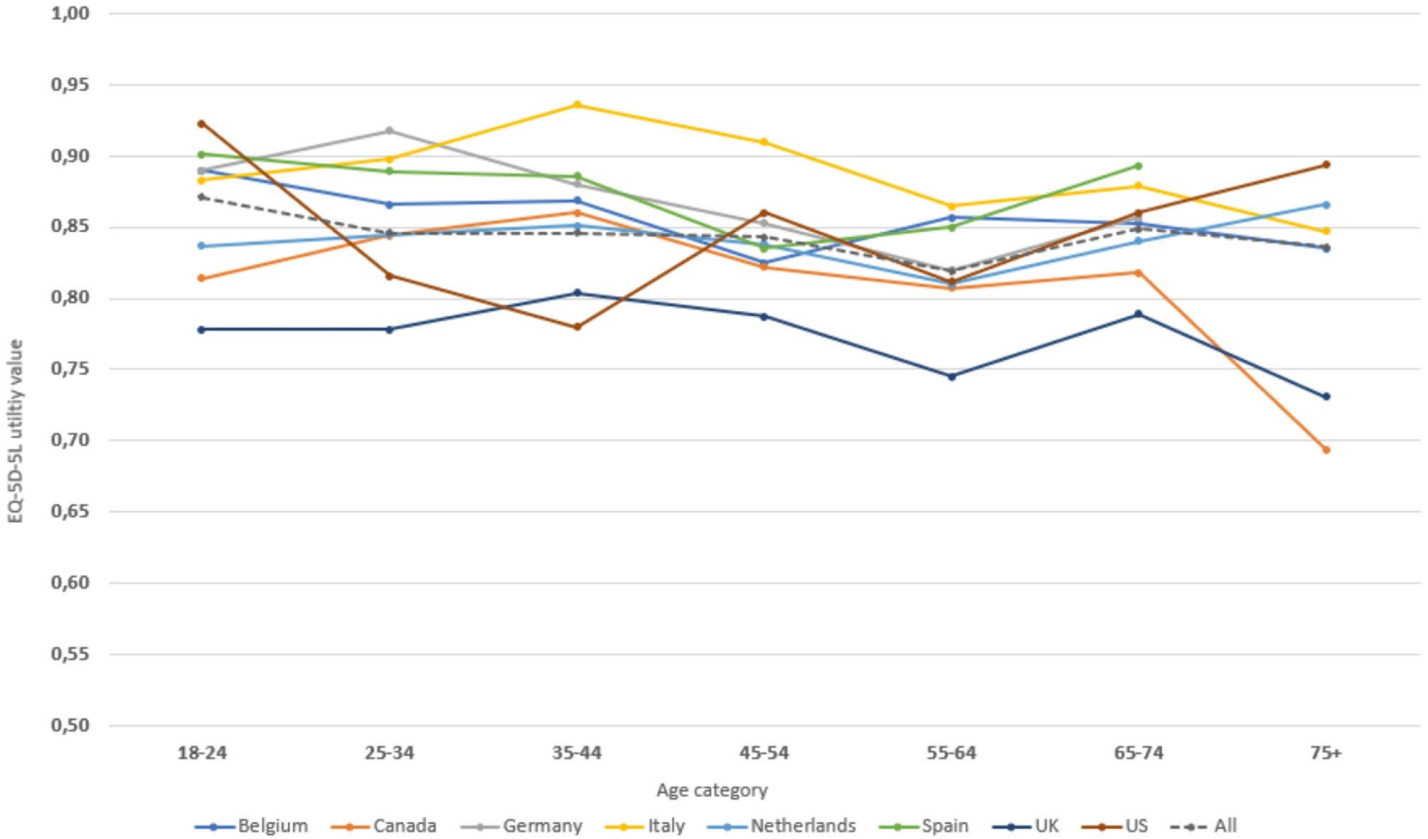
Plot of the mean EQ-5D-5L utility per country, by age category

Mean EQ-5D utility values are presented by age and sex in Table 4, per country and for the combined sample. The distribution of utility values by country is shown in Figure S1. The highest utility values were found in Italy, where especially men had much higher utilities (> 0.890), whilst the lowest utility values were found in the UK. Notably low utility values were found in Canadian and British men over 75 years of age (0.629 and 0.654 respectively), strongly contrasting the utility values found in Canadian and British women of the same age (0.775 and 0.781). The GLM models revealed that age, sex, and country have a statistically significant impact on utility values (all *p* < 0.0001), and the impact of age by sex is country-specific. For example, the sex effect was only consistent in Belgium, the Netherlands, and Italy (all *p* < 0.0001) with females having significantly lower utility values than males (Online Supplemental Material Table S4). The effect of sex was much smaller and not statistically significant in Canada, Germany, Spain, and in most age groups in the UK, with mean utility differences between males and females around 0.00-0.01. The effect of age was ambivalent: utility values decreased slightly with higher age but surged in some countries in people aged 65 years and older (Fig. 1). The highest utility values were sometimes observed in the youngest age group (this was the case in Belgium, Spain, US) or in the second youngest (Germany), but also sometimes in the middle (Canada, UK, Italy) or the highest age group (Netherlands). A regression analysis indicated that the interaction effect (effect of sex separately in each age groups) was either non-existent or limited.

**Table 4:** Population norms for the EQ-5D-5L utilities using country-specific value sets, and EQ VAS

	Belgium	Canada	Germany	Italy	Netherlands	Spain	UK	US	All countries
Sample size	*N* = 1000	*N* = 1000	*N* = 1000	*N* = 1000	*N* = 1000	*N* = 1000	*N* = 1000	*N* = 2000	*N* = 9000
**Female**	**Mean**, **SD** **(Q1; Q3)**	**Mean**, **SD** **(Q1; Q3)**	**Mean**, **SD** **(Q1; Q3)**	**Mean**, **SD** **(Q1; Q3)**	**Mean**, **SD** **(Q1; Q3)**	**Mean**, **SD** **(Q1; Q3)**	**Mean**, **SD** **(Q1; Q3)**	**Mean**, **SD** **(Q1; Q3)**	**Mean**, **SD** **(Q1; Q3)**
18–24	0.853, 0.105 (0.771; 0.919)	0.817, 0.172 (0.713; 0.922)	0.868, 0.193 (0.887; 0.970)	0.880, 0.096 (0.844; 0.956)	0.796, 0.266 (0.742; 1)	0.895, 0.075 (0.841; 1)	0.696, 0.285 (0.584; 0.879)	0.918, 0.261 (0.875; 1)	0.852, 0.175 (0.797; 1)
25–34	0.833, 0.166 (0.755; 1)	0.851, 0.141 (0.794; 1)	0.911, 0.083 (0.882; 0.907)	0.906, 0.080 (0.859; 1)	0.830, 0.23 3 (0.731; 1)	0.888, 0.089 (0.841; 1)	0.788, 0.306 (0.750; 1)	0.796, 0.645 (0.678; 1)	0.835, 0.243 (0.782; 1)
35–44	0.867, 0.177 (0.838; 1)	0.867, 0.147 (0.818; 1)	0.883, 0.170 (0.882; 1)	0.929, 0.127 (0.909; 1)	0.802, 0.230 (0.705; 1)	0.885, 0.150 (0.797; 1)	0.823, 0.216 (0.768; 1)	0.870, 0.243 (0.817; 1)	0.867, 0.191 (0.837; 1)
45–54	0.781, 0.255 (0.711; 0.922)	0.820, 0.221 (0.750; 1)	0.805, 0.205 (0.813; 1)	0.891, 0.234 (0.902; 1)	0.807, 0.211 (0.773; 0.918)	0.822, 0.242 (0.757; 0.922)	0.784, 0.211 (0.740; 1)	0.843, 0.248 (0.777; 1)	0.827, 0.230 (0.768; 1)
55–64	0.837, 0.149 (0.794; 1)	0.793, 0.180 (0.671; 0.922)	0.837, 0.296 (0.783; 0.943)	0.821, 0.190 (0.795; 0.953)	0.783, 0.215 (0.727; 0.887)	0.852, 0.324 (0.818; 0.922)	0.737, 0.281 (0.654; 0.879)	0.815, 0.173 (0.721; 1)	0.815, 0.214 (0.751; 0.943)
65–74	0.847, 0.107 (0.794; 0.922)	0.812, 0.176 (0.719; 1)	0.860, 0.276 (0.861; 0.970)	0.861, 0.160 (0.817; 0.953)	0.828, 0.217 (0.791; 1)	0.919, 0.000 (0.919; 0.919)	0.797, 0.252 (0.735; 1)	0.844, 0.152 (0.776; 1)	0.839, 0.174 (0.780; 0.956)
75+	0.821, 0.133 (0.757; 0.922)	0.775, 0.161 (0.652; 0.899)	.	0.804, 0.303 (0.793; 0.953)	0.815, 0.194 (0.765; 0.887)	.	0.781, 0.178 (0.708; 0.768)	0.928, 0.049 (0.844; 1)	0.817, 0.180 (0.756; 0.953)
All	0.832, 0.167 (0.771; 0.922)	0.827, 0.178 (0.757; 0.956)	0.862, 0.196 (0.825; 0.97)	0.874, 0.164 (0.844; 0.956)	0.812, 0.222 (0.743; 1)	0.860, 0.178 (0.818; 1)	0.771, 0.255 (0.708; 1)	0.840, 0.250 (0.779; 1)	0.835, 0.209 (0.778; 1)
**Male**									
18–24	0.947, 0.127 (0.922; 1)	0.811, 0.220 (0.663; 1)	0.912, 0.080 (0.877; 1)	0.887, 0.119 (0.844; 0.956)	0.879, 0.214 (0.883; 1)	0.909, 0.090 (0.872; 1)	0.851, 0.234 (0.729; 1)	0.929, 0.265 (0.940; 1)	0.891, 0.158 (0.844; 1)
25–34	0.894, 0.195 (0.797; 1)	0.837, 0.215 (0.785; 1)	0.925, 0.076 (0.877; 1)	0.892, 0.124 (0.859; 1)	0.860, 0.229 (0.791; 1)	0.890, 0.099 (0.841; 1)	0.767, 0.349 (0.717; 1)	0.835, 0.444 (0.810; 1)	0.857, 0.235 (0.794; 1)
35–44	0.872, 0.133 (0.841; 1)	0.853, 0.130 (0.771; 1)	0.877, 0.161 (0.861; 1)	0.942, 0.080 (0.909; 1)	0.905, 0.167 (0.883; 1)	0.887, 0.135 (0.841; 1)	0.788, 0.218 (0.750; 1)	0.717, 0.363 (0.630; 1)	0.827, 0.246 (0.791; 1)
45–54	0.858, 0.185 (0.818; 1)	0.824, 0.179 (0.713; 1)	0.857, 0.170 (0.835; 0.970)	0.931, 0.082 (0.903; 1)	0.867, 0.167 (0.817; 1)	0.849, 0.182 (0.794; 1)	0.792, 0.203 (0.698; 1)	0.886, 0.168 (0.844; 1)	0.859, 0.172 (0.796; 1)
55–64	0.884, 0.088 (0.838; 0.922)	0.825, 0.184 (0.794; 1)	0.800, 0.321 (0.733; 0.943)	0.931, 0.054 (0.902; 1)	0.861, 0.142 (0.817; 1)	0.847, 0.262 (0.818; 1)	0.754, 0.242 (0.664; 1)	0.807, 0.191 (0.738; 1)	0.824, 0.207 (0.771; 1)
65–74	0.859, 0.133 (0.823; 0.922)	0.823, 0.148 (0.757; 0.922)	0.854, 0.257 (0.867; 1)	0.899, 0.099 (0.858; 1)	0.852, 0.137 (0.808; 1)	0.884, 0.138 (0.798; 1)	0.777, 0.178 (0.735; 0.879)	0.876, 0.100 (0.817; 1)	0.860, 0.128 (0.806; 1)
75+	0.849, 0.144 (0.794; 1)	0.629, 0.354 (0.333; 0.922)	.	0.896, 0.135 (0.861; 1)	0.905, 0.088 (0.848; 1)	.	0.654, 0.180 (0.395; 0.721)	0.870, 0.076 (0.806; 0.94)	0.852, 0.161 (0.817; 1)
All	0.875, 0.153 (0.823; 1)	0.827, 0.184 (0.756; 1)	0.860, 0.183 (0.835; 1)	0.915, 0.096 (0.891; 1)	0.871, 0.163 (0.817; 1)	0.870, 0.165 (0.838; 1)	0.782, 0.236 (0.703; 1)	0.828, 0.265 (0.776; 1)	0.850, 0.197 (0.794; 1)
**All**									
18–24	0.890, 0.121 (0.794; 1)	0.814, 0.192 (0.713; 0.956)	0.890, 0.143 (0.877; 1)	0.883, 0.104 (0.844; 0.956)	0.837, 0.250 (0.778; 1)	0.901, 0.081 (0.841; 1)	0.778, 0.279 (0.635; 1)	0.923, 0.260 (0.883; 1)	0.871, 0.170 (0.817; 1)
25–34	0.866, 0.181 (0.771; 1)	0.844, 0.177 (0.794; 1)	0.918, 0.079 (0.882; 1)	0.898, 0.103 (0.859; 1)	0.845, 0.231 (0.778; 1)	0.889, 0.095 (0.841; 1)	0.778, 0.323 (0.750; 1)	0.816, 0.524 (0.743; 1)	0.846, 0.239 (0.794; 1)
35–44	0.869, 0.158 (0.841; 1)	0.860, 0.139 (0.794; 1)	0.880, 0.164 (0.877; 1)	0.936, 0.101 (0.909; 1)	0.851, 0.209 (0.782; 1)	0.886, 0.140 (0.841; 1)	0.804, 0.217 (0.768; 1)	0.780, 0.341 (0.701; 1)	0.846, 0.227 (0.811; 1)
45–54	0.825, 0.219 (0.757; 1)	0.822, 0.200 (0.730; 1)	0.853, 0.188 (0.829; 0.97)	0.910, 0.162 (0.902; 1)	0.838, 0.192 (0.782; 1)	0.835, 0.208 (0.794; 1)	0.787, 0.207 (0.703; 1)	0.860, 0.213 (0.777; 1)	0.843, 0.202 (0.776; 1)
55–64	0.857, 0.128 (0.815; 1)	0.807, 0.182 (0.751; 0.922)	0.820, 0.311 (0.783; 0.943)	0.865, 0.134 (0.844; 0.956)	0.810, 0.188 (0.743; 1)	0.850, 0.282 (0.818; 1)	0.745, 0.258 (0.654; 1)	0.812, 0.181 (0.738; 1)	0.819, 0.210 (0.766; 1)
65–74	0.853, 0.121 (0.797; 0.922)	0.818, 0.162 (0.756; 0.922)	0.857, 0.262 (0.861; 0.970)	0.879, 0.124 (0.844; 0.956)	0.840, 0.177 (0.804; 1)	0.893, 0.129 (0.838; 1)	0.789, 0.214 (0.735; 1)	0.860, 0.125 (0.806; 1)	0.849, 0.150 (0.794; 1)
75+	0.835, 0.139 (0.794; 0.922)	0.694, 0.303 (0.606; 0.899)	.	0.847, 0.193 (0.804; 0.956)	0.866, 0.139 (0.787; 1)	.	0.731, 0.185 (0.683; 0.735)	0.894, 0.070 (0.844; 1)	0.836, 0.168 (0.785; 1)
All	0.853, 0.162 (0.794; 1)	0.827, 0.181 (0.756; 1)	0.861, 0.189 (0.831; 1)	0.893, 0.131 (0.858; 1)	0.841, 0.196 (0.782; 1)	0.865, 0.171 (0.835; 1)	0.776, 0.245 (0.708; 1)	0.834, 0.259 (0.777; 1)	0.843, 0.203 (0.791; 1)
**EQ VAS**									
Mean	76.2	73.9	76.8	76.1	76.9	76.9	72.3	79.0	76.3
SD	15.6	17.7	18.5	15.9	16.1	16.4	19.1	18.1	17.4
Q1	70	66	70	70	70	70	61	70	70
Q3	88	87	90	90	90	90	88	91	90

Other factors significantly affecting utility values were place of residence, education, employment, and suffering from health conditions (all *p* < 0.0001). Participants living independently at home or with a family member had about twice the utility value compared to participants who lived at home but needed help from a caregiver, or who were living in a long-term care rehabilitation facility / in a nursing home (See Online Supplemental Material Table S2). Higher education was also associated with higher utility values and being in employment or studying lead to higher utility values than being unemployed, at home, or retired. Sick leave was also strongly associated with lower utility values (-0.300). All health conditions, except cancer (-0.181), were strongly associated with decreased utility values compared to those without health conditions, but this was especially observed in patients with dementia (-0.494), hemiplegia (-0.469), systemic lupus erythematosus (-0.455), congestive heart failure (-0.336), multiple sclerosis (-0.312), lupus nephritis (-0.309), psoriatic arthritis (-0.300), depression (-0.296), peptic ulcer disease (-0.281), and liver disease (-0.270).

### EQ VAS scores

**Fig. 2:**
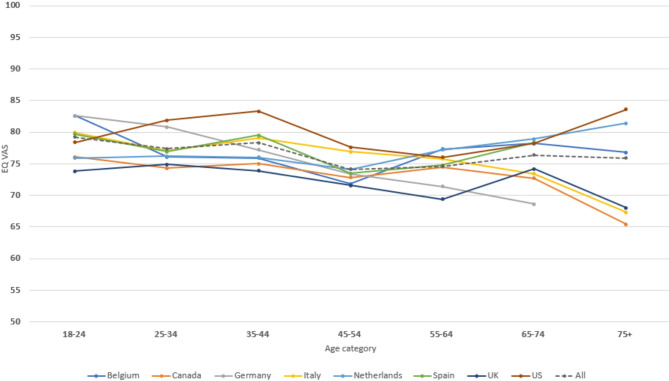
Plot of EQ VAS scores per age category, by country

The EQ VAS results are presented in Table 4, and the mean scores are compared to published national statistics in Table 1. The distribution of EQ VAS scores by country is shown in Figure S1. For most countries, the mean EQ VAS score in the POPUP study was similar to that reported in national statistics, with an overall mean EQ VAS score of 76.3. The Netherlands and the UK were exceptions: in these two countries, the POPUP EQ VAS scores were comparable to those in the other countries but considerably lower than their respective national statistics. Figure 2 shows the relationship between age and the EQ VAS score, which displays a fluctuating curve with peaks around 40 and 70 years of age, and dips around 30 and 60 years. The averaged EQ VAS across all countries declined between ages 18–45, and slightly increase between 45 and 75. VAS scores were similar between the sexes (75 female, 76 males) and that was true for most age groups. Correlations between EQ VAS and country-specific utility values were generally moderate, averaging around 0.56– with some variation, including a lower correlation in the US (0.39) and a higher value in Germany (0.73). When stratified by age groups, correlations were lower among individuals aged 18–44 (around 0.45) and higher among those aged 45–75 (around 0.65), suggesting that younger adults may value aspects of HRQoL not fully captured by the EQ-5D-5-L dimensions. A similar pattern was observed by sex, with a lower correlation in males (0.49) compared to females (0.63).

### HUI-3 dimension responses

Distribution of responses for all HUI-3 dimensions is presented in Table 5. The proportion of participants reporting “no problems” on all eight HUI-3 dimensions followed the same patterns as for the EQ-5D and was highest in the US (18.7%) and the Netherlands (13.0%), and lowest in Belgium (7.5%) and Germany (8.9%). Across all countries, reporting severe problems or being “unable to” perform an action in any HUI-3 dimension (last two levels) was most frequently observed in the dimension of pain (5.7%), followed by emotion (5.4%), and cognition (4.1%), whereas extreme problems or being “unable to” perform an action in a dimension was the least reported with dexterity (0.7%), followed by ambulation (1.2%). Severe problems or being “unable to” perform an action in any HUI-3 dimension were most frequently observed in the UK (18.1%), Canada (14.9%) and Germany (14.9%), and the least in the Netherlands (10.8%) and Italy (11.4%), which is comparable to the EQ-5D findings.

**Table 5:** Distribution of the respondents across the 8 domains of the HUI-3

Domains	Belgium	Canada	Germany	Italy	Netherlands	Spain	UK	US	All countries
Sample size	*N* = 1000	*N* = 1000	*N* = 1000	*N* = 1000	*N* = 1000	*N* = 1000	*N* = 1000	*N* = 2000	*N* = 9000
**Vision**									
Able to see well enough to read ordinary newsprint and recognize a friend on the other	29.6%	35.3%	32.5%	32.6%	33.5%	32.5%	36.2%	38.8%	34.4%
side of the street, without glasses or contact lenses.									
Able to see well enough to read ordinary newsprint and recognize a friend on the other	66.3%	58.8%	60.8%	62.6%	63.7%	63.2%	59.4%	55.6%	60.7%
side of the street, but with glasses.									
Able to read ordinary newsprint with or without glasses but unable to recognize a friend	1.3%	1.2%	1.8%	1.3%	1.5%	1.0%	1.5%	1.9%	1.5%
on the other side of the street, even with glasses.									
Able to recognize a friend on the other side of the street with or without glasses but	1.6%	2.3%	1.7%	1.3%	0.8%	2.3%	1.5%	1.6%	1.6%
unable to read ordinary newsprint, even with glasses.									
Unable to read ordinary newsprint and unable to recognize a friend on the other side of	0.9%	1.9%	2.4%	1.3%	0.5%	0.7%	0.6%	1.0%	1.2%
the street, even with glasses.									
Unable to see at all.	0.2%	0.6%	0.7%	0.9%	0.1%	0.3%	0.8%	1.2%	0.7%
**Hearing**									
Able to hear what is said in a group conversation with at least three other people, without	91.2%	90.6%	94.1%	92.2%	90.2%	94.6%	88.8%	84.1%	90.0%
a hearing aid.									
Able to hear what is said in a conversation with one other person in a quiet room without	1.2%	1.1%	0.2%	0.7%	1.2%	1.1%	1.5%	2.8%	1.4%
a hearing aid, but requires a hearing aid to hear what is said in a group conversation with at									
least three other people.									
Able to hear what is said in a conversation with one other person in a quiet room with a	2.9%	4.0%	1.7%	3.0%	4.8%	1.4%	3.8%	6.1%	3.8%
hearing aid and able to hear what is said in a group conversation with at least three other									
people with a hearing aid.									
Able to hear what is said in a conversation with one other person in a quiet room without	2.5%	1.3%	1.2%	1.3%	1.8%	1.4%	2.8%	2.2%	1.8%
a hearing aid, but unable to hear what is said in a group conversation with at least three other									
people even with a hearing aid.									
Able to hear what is said in a conversation with one other person in a quiet room with a	1.0%	0.5%	0.3%	0.5%	1.2%	0.3%	0.6%	1.4%	0.8%
hearing aid, but unable to hear what is said in a group conversation with at least three other									
people even with a hearing aid.									
Unable to hear at all.	1.1%	2.6%	2.4%	2.4%	0.9%	1.3%	2.5%	3.4%	2.2%
**Speech**									
Able to be understood completely when speaking with strangers or friends.	93.9%	92.5%	93.7%	94.1%	93.2%	93.8%	92.8%	86.2%	91.8%
Able to be understood partially when speaking with strangers but able to be understood	2.9%	2.0%	2.8%	1.6%	2.6%	3.0%	2.6%	4.2%	2.9%
completely when speaking with people who know me well.									
Able to be understood partially when speaking with strangers or people who know me	2.6%	3.2%	2.2%	3.2%	3.1%	2.0%	3.0%	6.3%	3.5%
well.									
Unable to be understood when speaking with strangers but able to be understood	0.4%	0.5%	0.4%	0.1%	0.6%	0.9%	0.3%	1.8%	0.7%
partially by people who know me well.									
Unable to be understood when speaking to other people (or unable to speak at all).	0.2%	1.8%	0.8%	1.0%	0.5%	0.3%	1.3%	1.7%	1.0%
**Ambulation**									
Able to walk around the neighborhood without difficulty, and without walking equipment.	85.8%	81.4%	82.7%	87.5%	83.9%	89.0%	78.9%	78.0%	82.8%
Able to walk around the neighborhood with difficulty, but does not require walking	10.4%	12.2%	12.1%	7.9%	9.0%	8.5%	11.3%	13.3%	10.9%
equipment or the help of another person.									
Able to walk around the neighborhood with walking equipment, but without the help of	1.9%	3.2%	3.6%	2.9%	4.5%	1.2%	4.0%	5.0%	3.5%
another person.									
Able to walk only short distances with walking equipment, and requires a wheelchair to	1.1%	1.9%	1.0%	0.4%	1.3%	0.3%	3.6%	2.7%	1.7%
get around the neighborhood.									
Unable to walk alone, even with walking equipment. Able to walk short distances with the	0.5%	0.6%	0.2%	1.2%	0.5%	0.8%	1.4%	0.7%	0.7%
help of another person, and requires a wheelchair to get around the neighborhood.									
Able to walk around the neighborhood without difficulty, and without walking equipment.	0.3%	0.7%	0.6%	0.2%	0.8%	0.2%	0.8%	0.3%	0.5%
**Dexterity**									
Full use of two hands and ten fingers.	89.2%	88.2%	88.8%	91.5%	89.8%	92.3%	88.6%	84.4%	88.6%
Limitations in the use of hands or fingers, but does not require special tools or help of	7.1%	7.7%	6.5%	5.6%	6.3%	4.7%	6.9%	7.6%	6.7%
another person.									
Limitations in the use of hands or fingers, is independent with use of special tools (does	1.5%	2.2%	2.6%	1.6%	1.9%	1.3%	1.9%	4.4%	2.4%
not require the help of another person).									
Limitations in the use of hands or fingers, requires the help of another person for some	1.8%	1.4%	1.6%	1.2%	1.4%	1.2%	1.3%	2.7%	1.7%
tasks (not independent even with the use of special tools).									
Limitations in the use of hands or fingers, requires the help of another person for most	0.4%	0.2%	0.3%	0.1%	0.5%	0.2%	0.8%	0.5%	0.4%
tasks (not independent even with the use of special tools).									
Limitations in the use of hands or fingers, requires the help of another person for all tasks	0.1%	0.4%	0.3%	0.1%	0.1%	0.3%	0.6%	0.5%	0.3%
(not independent even with the use of special tools).									
**Emotion**									
Happy and interested in life.	37.7%	39.5%	33.2%	28.4%	52.2%	31.9%	31.8%	58.0%	41.2%
Somewhat happy.	44.9%	36.4%	46.9%	50.7%	31.8%	45.8%	43.1%	26.3%	39.1%
Somewhat unhappy.	13.1%	17.7%	14.2%	17.2%	13.3%	15.6%	17.2%	10.5%	14.4%
Very unhappy.	3.2%	3.9%	4.2%	2.7%	1.7%	5.3%	4.5%	3.8%	3.7%
So unhappy that life is not worthwhile.	1.2%	2.5%	1.5%	1.0%	1.1%	1.5%	3.4%	1.5%	1.7%
**Cognition**									
Able to remember most things, think clearly and solve day to day problems.	70.2%	73.6%	73.3%	81.5%	77.4%	70.8%	67.3%	72.4%	73.2%
Able to remember most things, but have a little difficulty when trying to think and solve	7.3%	4.4%	4.5%	5.1%	8.0%	6.2%	5.1%	3.3%	5.3%
day to day problems.									
Somewhat forgetful, but able to think clearly and solve day to day problems.	9.6%	8.6%	10.1%	6.3%	3.4%	10.9%	13.5%	10.3%	9.2%
Somewhat forgetful, and have a little difficulty when trying to think or solve day to day	9.7%	9.0%	9.4%	5.8%	8.0%	8.4%	9.1%	7.5%	8.3%
problems.									
Very forgetful, and have great difficulty when trying to think or solve day to day problems.	2.4%	4.0%	2.5%	1.1%	2.2%	3.4%	4.3%	4.6%	3.2%
Unable to remember anything at all, and unable to think or solve day to day problems.	0.7%	0.4%	0.2%	0.3%	1.1%	0.4%	0.6%	1.9%	0.8%
**Pain**									
Free of pain and discomfort.	37.5%	43.9%	34.8%	47.5%	51.9%	42.9%	45.9%	50.1%	45.0%
Mild to moderate pain that prevents no activities.	42.6%	39.4%	45.2%	42.0%	29.9%	42.4%	34.1%	34.1%	38.2%
Moderate pain that prevents a few activities.	14.2%	12.8%	13.0%	7.9%	13.2%	8.1%	10.8%	10.0%	11.1%
Moderate to severe pain that prevents some activities.	4.9%	2.5%	5.0%	1.9%	4.2%	4.4%	4.8%	4.4%	4.1%
Severe pain that prevents most activities.	0.8%	1.5%	2.0%	0.7%	0.8%	2.3%	4.4%	1.3%	1.7%
**Overall**									
% no problems in every dimension	7.5%	11.3%	8.9%	9.5%	13.0%	9.1%	9.7%	18.7%	11.8%
% severe problems or “unable to” in at least one dimension	11.7%	14.9%	14.9%	11.4%	10.8%	13.2%	18.1%	16.8%	14.3%
% negative utility values	2.4%	4.1%	2.7%	2.1%	2.5%	3.1%	4.6%	5.6%	3.6%

Ocular problems of any severity were most frequently reported in the Belgian population (70.4%) and least frequently in the US (61.2%). However, the percentage of people who could not see at all was highest in the US (1.2%), and is relatively high compared to Belgium, the Netherlands and Spain (< 0.4%). Problems with hearing were most prevalent in the US (15.9%,), approximately three times as much as in Germany and Spain, and twice the rate measured in Italy. In addition, 3.4% of the American sample could not hear at all, whilst this was only up to 2.5% of the population in the other countries. Problems with speech were also most frequently observed in the US (13.8%), while this was on average 6.6% in other research countries. Approximately one-fifth of participants in the UK (21.1%), US (22.0%) and Canada (18.6%) reported ambulation problems of any severity, which is almost twice as much as in Spain (11%) and Italy (12.6%). Approximately 90% of participants had no dexterity problems, although this percentage was slightly higher in Italy and Spain, and the lowest in the US.

### HUI-3 utility values

Mean HUI-3 utility values for all countries combined ranged from 0.717 in participants between 25 and 34 years of age to 0.767 and 0.768 in participants aged 35–44 and 65–74 years, respectively (Table 6). Overall, highest utility values were observed in the Netherlands (0.779), closely followed by Italy (0.778), and were lowest in the in the UK (0.706).

**Table 6:** Population norms for the HUI-3 utilities, using the Canadian value set

	Belgium	Canada	Germany	Italy	Netherlands	Spain	UK	US	All countries
Sample size	*N* = 1000	*N* = 1000	*N* = 1000	*N* = 1000	*N* = 1000	*N* = 1000	*N* = 1000	*N* = 2000	*N* = 9000
**Female**	Mean, SD (Q1; Q3)	Mean, SD (Q1; Q3)	Mean, SD (Q1; Q3)	Mean, SD (Q1; Q3)	Mean, SD (Q1; Q3)	Mean, SD (Q1; Q3)	Mean, SD (Q1; Q3)	Mean, SD (Q1; Q3)	Mean, SD (Q1; Q3)
18–24	0.780, 0.160 (0.69; 0.91)	0.732, 0.316 (0.58; 0.93)	0.774, 0.193 (0.68; 0.92)	0.730, 0.209 (0.66; 0.93)	0.743, 0.298 (0.64; 0.97)	0.798, 0.139 (0.69; 0.92)	0.623, 0.355 (0.38; 0.88)	0.767, 0.487 (0.57; 0.97)	0.749, 0.243 (0.65; 0.93)
25–34	0.699, 0.327 (0.63; 0.93)	0.757, 0.251 (0.64; 0.95)	0.790, 0.175 (0.73; 0.93)	0.780, 0.149 (0.68; 0.93)	0.799, 0.272 (0.76; 0.95)	0.753, 0.175 (0.66; 0.92)	0.714, 0.368 (0.71; 0.92)	0.688, 0.840 (0.54; 1.00)	0.734, 0.337 (0.65; 0.97)
35–44	0.802, 0.234 (0.73; 0.93)	0.797, 0.261 (0.77; 0.97)	0.758, 0.234 (0.68; 0.92)	0.841, 0.181 (0.78; 0.95)	0.730, 0.301 (0.57; 0.97)	0.731, 0.343 (0.55; 0.93)	0.733, 0.266 (0.68; 0.93)	0.777, 0.370 (0.66; 1.00)	0.774, 0.282 (0.68; 0.97)
45–54	0.689, 0.287 (0.54; 0.91)	0.712, 0.300 (0.66; 0.93)	0.763, 0.246 (0.68; 0.93)	0.784, 0.234 (0.73; 0.91)	0.747, 0.275 (0.64; 0.95)	0.700, 0.362 (0.58; 0.93)	0.694, 0.271 (0.51; 0.91)	0.730, 0.359 (0.66; 0.95)	0.728, 0.298 (0.64; 0.92)
55–64	0.783, 0.176 (0.72; 0.92)	0.739, 0.217 (0.60; 0.92)	0.696, 0.472 (0.57; 0.91)	0.716, 0.222 (0.64; 0.85)	0.767, 0.212 (0.70; 0.92)	0.761, 0.502 (0.73; 0.91)	0.641, 0.368 (0.46; 0.91)	0.726, 0.251 (0.57; 0.97)	0.724, 0.293 (0.62; 0.92)
65–74	0.745, 0.185 (0.66; 0.91)	0.731, 0.226 (0.66; 0.92)	0.693, 0.475 (0.55; 0.91)	0.790, 0.199 (0.73; 0.91)	0.756, 0.226 (0.62; 0.92)	0.821, 0.067 (0.79; 0.84)	0.709, 0.284 (0.62; 0.91)	0.765, 0.189 (0.70; 0.92)	0.752, 0.219 (0.67; 0.92)
75+	0.683, 0.244 (0.56; 0.91)	0.704, 0.141 (0.62; 0.82)	.	0.709, 0.413 (0.63; 0.91)	0.739, 0.232 (0.59; 0.92)	.	0.696, 0.248 (0.53; 0.73)	0.844, 0.168 (0.74; 1.00)	0.720, 0.257 (0.62; 0.92)
All	0.739, 0.243 (0.65; 0.92)	0.745, 0.259 (0.65; 0.93)	0.739, 0.281 (0.64; 0.91)	0.771, 0.211 (0.72; 0.92)	0.756, 0.260 (0.64; 0.95)	0.749, 0.291 (0.68; 0.92)	0.684, 0.317 (0.54; 0.91)	0.738, 0.346 (0.63; 0.97)	0.740, 0.283 (0.65; 0.93)
**Male**									
18–24	0.872, 0.381 (0.88; 1.00)	0.632, 0.431 (0.47; 0.93)	0.762, 0.217 (0.73; 0.93)	0.741, 0.300 (0.66; 0.93)	0.853, 0.338 (0.85; 0.97)	0.803, 0.206 (0.78; 0.95)	0.786, 0.362 (0.68; 1.00)	0.769, 0.571 (0.59; 1.00)	0.773, 0.318 (0.70; 0.97)
25–34	0.743, 0.378 (0.58; 0.97)	0.700, 0.378 (0.56; 0.97)	0.776, 0.195 (0.69; 0.97)	0.670, 0.315 (0.45; 0.93)	0.759, 0.415 (0.68; 1.00)	0.775, 0.191 (0.71; 0.93)	0.719, 0.438 (0.64; 0.93)	0.614, 0.557 (0.25; 0.95)	0.702, 0.361 (0.55; 0.95)
35–44	0.793, 0.227 (0.68; 0.97)	0.784, 0.215 (0.67; 0.93)	0.761, 0.253 (0.68; 0.97)	0.795, 0.223 (0.75; 0.97)	0.836, 0.246 (0.79; 1.00)	0.824, 0.189 (0.75; 0.97)	0.701, 0.305 (0.62; 0.95)	0.714, 0.308 (0.59; 1.00)	0.762, 0.265 (0.67; 0.97)
45–54	0.747, 0.263 (0.67; 0.92)	0.710, 0.290 (0.57; 0.95)	0.753, 0.231 (0.69; 0.92)	0.833, 0.172 (0.77; 0.97)	0.804, 0.269 (0.75; 0.97)	0.769, 0.235 (0.68; 0.95)	0.760, 0.241 (0.69; 0.93)	0.775, 0.321 (0.72; 0.97)	0.770, 0.257 (0.71; 0.97)
55–64	0.806, 0.152 (0.71; 0.92)	0.790, 0.209 (0.77; 0.95)	0.702, 0.340 (0.58; 0.91)	0.865, 0.083 (0.84; 0.97)	0.822, 0.154 (0.77; 0.97)	0.707, 0.357 (0.61; 0.92)	0.701, 0.267 (0.56; 0.91)	0.745, 0.217 (0.60; 0.97)	0.744, 0.242 (0.65; 0.92)
65–74	0.779, 0.173 (0.70; 0.92)	0.744, 0.230 (0.55; 0.97)	0.781, 0.243 (0.68; 0.92)	0.792, 0.148 (0.73; 0.92)	0.790, 0.170 (0.67; 0.97)	0.792, 0.215 (0.72; 0.92)	0.769, 0.206 (0.75; 0.92)	0.804, 0.154 (0.73; 0.95)	0.785, 0.172 (0.70; 0.93)
75+	0.764, 0.188 (0.71; 0.92)	0.590, 0.381 (0.41; 0.85)	.	0.750, 0.168 (0.68; 0.91)	0.812, 0.160 (0.65; 0.97)	.	0.275, 0.341 (-0.15; 0.41)	0.678, 0.298 (0.52; 0.92)	0.732, 0.233 (0.63; 0.92)
All	0.770, 0.242 (0.69; 0.93)	0.727, 0.286 (0.60; 0.95)	0.746, 0.248 (0.66; 0.93)	0.786, 0.202 (0.75; 0.93)	0.803, 0.253 (0.74; 0.97)	0.767, 0.243 (0.70; 0.93)	0.728, 0.295 (0.65; 0.93)	0.724, 0.305 (0.59; 0.97)	0.753, 0.265 (0.66; 0.95)
**All**									
18–24	0.815, 0.230 (0.73; 0.97)	0.682, 0.369 (0.48; 0.93)	0.768, 0.206 (0.69; 0.93)	0.735, 0.241 (0.66; 0.93)	0.797, 0.319 (0.74; 0.97)	0.800, 0.168 (0.74; 0.93)	0.709, 0.370 (0.54; 0.93)	0.768, 0.528 (0.59; 1.00)	0.760, 0.276 (0.66; 0.95)
25–34	0.722, 0.347 (0.62; 0.93)	0.728, 0.314 (0.60; 0.97)	0.783, 0.186 (0.70; 0.95)	0.719, 0.247 (0.62; 0.93)	0.778, 0.345 (0.70; 0.97)	0.767, 0.185 (0.67; 0.93)	0.716, 0.397 (0.66; 0.93)	0.650, 0.673 (0.25; 1.00)	0.717, 0.350 (0.61; 0.97)
35–44	0.799, 0.230 (0.73; 0.95)	0.791, 0.239 (0.70; 0.95)	0.760, 0.245 (0.68; 0.93)	0.816, 0.208 (0.76; 0.95)	0.780, 0.281 (0.67; 1.00)	0.782, 0.253 (0.73; 0.97)	0.715, 0.288 (0.63; 0.95)	0.740, 0.328 (0.60; 1.00)	0.767, 0.272 (0.67; 0.97)
45–54	0.722, 0.274 (0.60; 0.92)	0.711, 0.294 (0.62; 0.95)	0.759, 0.238 (0.68; 0.92)	0.807, 0.200 (0.75; 0.93)	0.776, 0.273 (0.67; 0.97)	0.734, 0.295 (0.65; 0.95)	0.725, 0.259 (0.62; 0.92)	0.748, 0.341 (0.66; 0.97)	0.748, 0.278 (0.67; 0.93)
55–64	0.793, 0.166 (0.72; 0.92)	0.762, 0.214 (0.67; 0.92)	0.699, 0.401 (0.57; 0.91)	0.775, 0.165 (0.73; 0.92)	0.786, 0.189 (0.70; 0.92)	0.738, 0.408 (0.67; 0.92)	0.671, 0.311 (0.52; 0.91)	0.733, 0.237 (0.59; 0.97)	0.733, 0.268 (0.63; 0.92)
65–74	0.763, 0.179 (0.67; 0.92)	0.738, 0.227 (0.62; 0.93)	0.732, 0.366 (0.65; 0.92)	0.791, 0.167 (0.73; 0.91)	0.772, 0.197 (0.65; 0.95)	0.800, 0.200 (0.77; 0.91)	0.733, 0.246 (0.65; 0.92)	0.785, 0.170 (0.71; 0.95)	0.768, 0.194 (0.68; 0.92)
75+	0.725, 0.217 (0.59; 0.92)	0.641, 0.317 (0.55; 0.85)	.	0.728, 0.249 (0.68; 0.91)	0.780, 0.189 (0.64; 0.97)	.	0.532, 0.374 (0.41; 0.73)	0.746, 0.262 (0.52; 0.95)	0.727, 0.241 (0.62; 0.92)
All	0.754, 0.243 (0.67; 0.92)	0.736, 0.272 (0.62; 0.95)	0.742, 0.263 (0.66; 0.92)	0.778, 0.206 (0.73; 0.93)	0.779, 0.257 (0.68; 0.97)	0.758, 0.264 (0.68; 0.93)	0.706, 0.307 (0.59; 0.92)	0.731, 0.323 (0.60; 0.97)	0.746, 0.274 (0.66; 0.93)

**Fig. 3:**
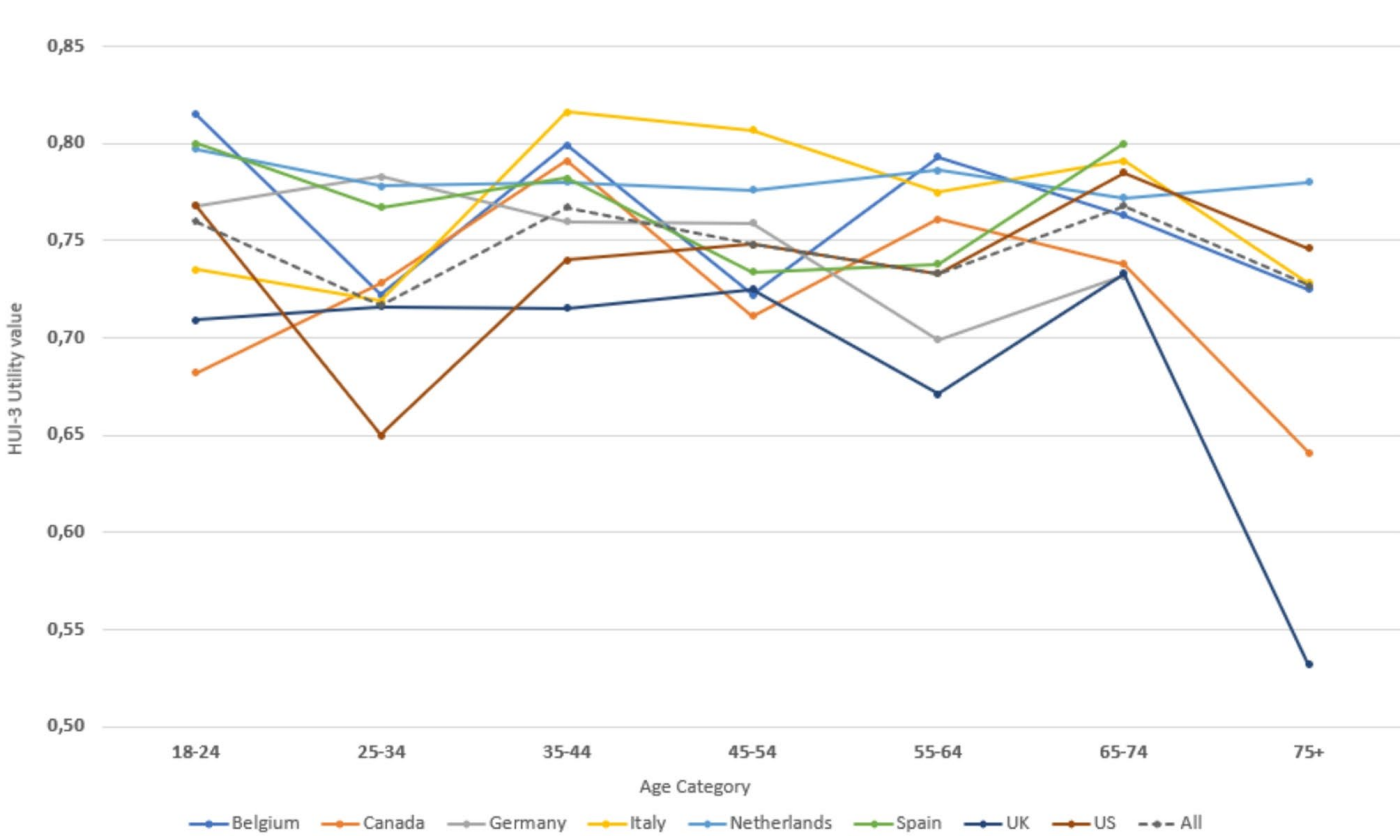
Plot of the mean HUI-3 utility per country, by age category

HUI-3 utility values did not show a specific trend by age but were rather fluctuating (Fig. 3). This variability was observed in all countries and in the total sample. Overall, no large differences were found between males and females (0.753 vs. 0.740), however the effect of sex varied across age groups and countries. Statistical inference confirmed that age, sex, and country each had a statistically significant impact independent of each other, and that the impact of age and sex differed by country. However, across the board, whilst statistically significant, the impact of sex was small.

Several other factors influenced HUI-3 utility values, as evidenced by the heterogeneous values across subgroups (Online Supplemental Material Table S3). HUI-3 utility values were significantly and markedly lower for people with primary (-0.111) or secondary education (-0.055) compared to those who had a higher degree; for people who are unemployed (-0.082), cannot work due to illness (-0.357) or homemakers (-0.051) compared to those who are actively working; for those who live at home but need help from a caregiver (-0.492) or live in a nursing home (-0.654) or in a long-term rehabilitation facility (-0.281) compared to the people who live at home independently; for those who were on sick leave during the past 4 weeks (-0.260); for those who need regular help from a caregiver (-0.429) compared to the people who don’t need a caregiver; and for those suffering from selected health conditions. The ten health conditions with the largest HUI-3 utility decrement were dementia (-0.749), hemiplegia (-0.705), systemic lupus erythematosus (-0.583), lupus nephritis (-0.500), congestive heart failure (-0.402), depression (-0.392), psoriatic arthritis (-0.382), HIV (-0.381), multiple sclerosis (-0.341), and Crohn’s disease (-0.336) (all *p* < 0.0001).

## Discussion

### Main results

This study presents EQ-5D-5-L and HUI-3 population norms for Belgium, Canada, Germany, Italy, the Netherlands, Spain, the UK, and the US. HRQoL data of 9,000 members of the general population across eight countries were collected with the aim of establishing an HRQoL baseline which can be used as reference for comparison across different (patient) populations. Important differences in EQ-5D-5-L utilities, EQ VAS scores, and HUI-3 utilities, were found between countries and subgroups defined by age and sex, highlighting the need for country-specific population norms.

The effect of sex differed by country and was in many countries smaller than the commonly accepted minimally important difference (MID); even though differences were statistically significant due to the large sample size. The MID for the EQ-5D-5-L has been heterogeneously reported, ranging from 0.040 (US) to 0.082 (UK) [40], or from 0.037 (Canada and England) to 0.058 (China) [41]. The MID for the HUI-3 has been estimated to be 0.03, although some studies consider a difference of 0.01 to be important [42]. Differences between males and females were often smaller (0.00-0.01) except in Belgium and the Netherlands. The effect of age on utility values was not consistent across instruments and countries. In some cases, age-related trends diverged considerably between EQ-5D-5-L, EQ VAS, and HUI-3 scores highlighting that they may capture different aspects of HRQoL. Although differences in utility values by age were statistically significant, they were often small. Hence, interpreting these trends warrants consideration of the MID for each HRQoL measure.

### Comparison with published literature

A publication of EQ-5D-5-L population norms for 23 countries also showed a more linear effect of age, with utility values steadily decreasing as age increased [10]. In contrast, in some countries, utility values and EQ VAS scores showed un upward trend among people aged between 55 and 74 (e.g., Canada and the US), as observed in our study [10]. Lubetkin et al. similarly found EQ-5D-5-L utility values increased with age in a representative panel survey of New Yorkers during the COVID-19 pandemic [43]. The higher utility values among respondents aged 65 and older in our study may reflect higher educational levels, which are associated with better health and self-perceived well-being after retirement [44]. Prior studies have shown similar patterns, attributing elevated HRQoL in older adults to education and generally good health status, particularly in the US and Netherlands [9, 45]. Females often reported slightly more problems on several dimensions, resulting in lower utility values than males, which has also been found in previously published population norms [10]. Pain/anxiety was the dimension with the highest prevalence of problems and self-care the least, which was also observed in POPUP [10]. When comparing our results with previously reported population norms, our EQ-5D-5-L utility values were slightly lower in most countries, and the proportion of participants reporting problems of any severity was higher. Although the association was not as strong as expected, participants with cancer reported utility values on average 0.181 lower than those without health conditions (range: 0.086–0.289), suggesting reduced HRQoL among cancer survivors. This was also the case for HUI-3 outcomes, and here differences were even larger. These differences with previously published population norms will be further explored in the following paragraphs, for both instruments.

#### EQ-5D norms per country

The proportions of Belgian respondents reporting problems of any severity were nearly identical to those reported by Van Wilder et al., in the general population of Belgium in 2018 [46]. The mean EQ-5D-5-L utility values measured in that study were similar to ours (van Wilder et al. = 0.840 and POPUP = 0.853) and were also significantly higher in males than in females.

When comparing our Canadian EQ-5D responses per dimension to a previously published population norms study based on the preferences of the Quebec population [47], the proportion of patients reporting no problems was similar for all dimensions except pain/discomfort (POPUP = 43.6% vs. Quebec = 32.1%). The mean utility values were comparable in both studies (POPUP = 0.827 vs. Quebec = 0.824), and to utility values measured in the Canadian region Alberta (0.840) [48]. Mean EQ VAS scores were lower (POPUP = 73.9, Quebec = 75.9, and Alberta = 77.4).

Four studies previously reported EQ-5D-5-L population norms for the German population, using data from 2011 [49], 2012–2014 [50], 2014 [51] and 2015 [52]. Like our study, the dimensions with the highest and lowest proportion of respondents reporting “no problems” were self-care and pain/discomfort respectively in all studies. However, the EQ-5D-5-L studies based on data from 2011 and aggregate data from 2012 to 2014 showed markedly higher proportions of participants reporting “no problems” on all dimensions compared to POPUP. The proportions of participants reporting “no problems” were relatively lower in our study for mobility (POPUP = 68.8% vs. 94.0%) [50], usual activities (POPUP = 72.5% vs. 86.8%) [50], pain/discomfort (POPUP = 36.0% vs. 54.4% [49]; 68.3% [50]; 71.2% [49]), and anxiety/depression (POPUP = 58.0% vs. 77.4% [49]; 82.1% [50]; 74.9% [51]. The utility value however was of similar magnitude (POPUP = 0.861 vs. 0.880) [51]. Self-perceived health with the EQ VAS was higher in our sample compared to the study based on data from 2014 (POPUP = 76.8 vs. 71.6) [51], while two other studies observed higher EQ VAS scores (84.3 [50]; 85.1 [52]).

The proportion of Spanish respondents in our study reporting “no problems” was similar for most dimensions in comparison with a Spanish EQ-5D population norms study with national health survey data from 2011–2012 [53], but smaller for the dimensions pain/discomfort (POPUP = 52.4% vs. 71.7%) and anxiety/depression (POPUP = 53.3% vs. 83.6%). This difference may be explained by the lower proportion of respondents reporting medical conditions in the POPUP study compared to the Spanish study (POPUP = 58.0% vs. 73.6%). While the POPUP study collected more detailed information on medical conditions, the previous study recorded the presence of at least one medical condition as a binary variable (yes/no). Despite these differences in the EQ-5D-5-L response distributions, utility values in the Spanish general population were comparable to our study (POPUP = 0.865 vs. 0.897), as were the mean EQ VAS scores (POPUP = 76.9 vs. 75.7) [53].

Similarly to EQ-5D-5-L population norms based on data collected in six American metropolitan areas in 2017 [9], self-care was the dimension with the fewest reported problems (POPUP = 12.0% vs. 6.5%), whilst pain/discomfort had the highest proportion of respondents reporting problems (POPUP 52.5% vs. 51.0%). The proportion of patients reporting no problems was similar to slightly higher in our study for all dimensions except self-care (POPUP = 88.0% vs. 93.5%) [9].

#### HUI-3

Age- and sex-specific Canadian utility standards, based on the Canadian Community Health Survey 2013–2014, reported mean (95% CI) HUI-3 utilities of all ages in the Canadian population of 0.863 (0.861–0.865) [54]. This is markedly, and significantly higher than the mean utility value of 0.736 found in our study for the adult general population of Canada, and this observation was consistently found across all age groups. Possible explanations of the difference are cohort effects (there is a 10-year difference in the data collection dates), the COVID-19 pandemic (which started eight months before the POPUP data collection and had not ended when collecting the data).

HUI-3 populations norms were also produced for the general adult US population based on the 2002 US Census [55]. A mean HUI-3 utility value of 0.81 was found in the US study, with notably higher values in individuals between 18 and 44 years of age (0.86). These values are markedly higher than the mean HUI-3 utility values of 0.731 we observed for the US adult population in POPUP. The fifteen years lag in these two data collections is certainly responsible for part of this difference; the COVID-19 pandemic could be for another part. Furthermore, the US study purposely oversampled on Hispanic and Black individuals, which were the respondents with the highest utility values. Furthermore, the US Census was conducted face to face, so any differences could also be due to the mode of administration. Comparison of HUI-3 utility scores across the countries included in our study is limited by the absence of country-specific HUI-3 value sets. Further research is needed to develop and validate these value sets to ensure more accurate and culturally relevant utility estimates for international comparisons.

#### EQ-5D-5-L bolt-ons

Bolt-ons to the EQ-5D-5-L are increasingly being developed and used [16]. However, comparing results across studies remain challenging, as most investigations are conducted in patient populations. To the best of our knowledge, only two studies have assessed bolt-ons similar to those used in our study within general population samples, specifically in Hungary and Australia [56, 57]. Compared to the POPUP sample, the Hungarian general population reported lower proportions of severe-to-extreme problems across all bolt-ons: vision (1.0%), breathing (0.0%), sleep (1.0%), tiredness (3.1%), self-confidence (5.0%), and social relationships (2.2%) [56]. In the Australian general population, where only the breathing bolt-on was evaluated, a comparable proportion of severe-to-extreme problems was reported (3.3%) [57]. Further research is needed to better understand how these bolt-ons perform across different populations and contexts, and to establish normative values that support their use in both clinical and population health settings [58].

### Strengths and limitations

#### Strengths

Data were collected among a large sample of the general population for two prominent HRQoL measures, in eight different countries. This study presents up to date normative HRQoL data which can be used for model-based economic evaluations for the estimation of QALYs and for comparison of specific patient groups with the general populations of all included countries. Only one mode of administration was used, increasing the comparability of population norms across countries.

#### Limitations

Several limitations should be considered when interpreting the results of this study and considering its implications for healthcare policy and practice. Our sample sizes (*N* = 2,000 in the US; *N* = 1,000 in other countries) yield margins of error of 3.2% and 2.2%, respectively—well below the commonly accepted 10% threshold [20]. Still, these margins should be considered when interpreting results, as samples may not fully reflect national populations. Differences in education levels, for example, could influence utility values and health profiles. Although sampling was stratified by age, sex, education, and region, no data were available on chronic conditions or treatment status at the recruitment stage, so the presence of cancer or other health conditions was not accounted for. This may partly explain the non-significant differences in HRQoL between cancer patients and healthy individuals. Selection bias is also possible, as the online survey excluded those without internet access. Additionally, the use of incentives may have attracted participants with lower socioeconomic status, affecting sample representativeness [59]. Furthermore, data were collected during the COVID-19 pandemic, which could have negatively impacted our outcomes and affect the external validity of our findings. According to a systematic review [60], the pandemic significantly affected all dimensions of the EQ-5D-5-L, and had negative impact on the HRQoL of the general population. A study on the impact of COVID-19 in the US concluded the same, and found that especially the mental health of young adults was impacted [61]. While these studies provide a viable explanation for disparities observed in, for example, the proportion of individuals reporting ‘no problems’, and varying anxiety/depression levels compared to previously published population norms, other literature does not support this reasoning. A longitudinal study found that the HRQoL of respondents in five different countries was not or only slightly deteriorated over the period of one year during the COVID-19 pandemic [62, 63]. Similarly, another study concluded that HRQoL collected during the COVID-19 pandemic can still confidently be used in clinical research [63]. Altogether, it remains difficult to pinpoint the exact impact of the COVID-19 pandemic on our data since this differs per country, and almost no data is available to explain its impact on bolt-ons and the HUI-3. Finally, while the HUI-3 and EQ-5D-5-L are widely used and validated measures of health status, they may not capture all aspects of health that are important to individuals or may not be sensitive enough to detect subtle differences in health status between individuals.

## Conclusion

With this multinational digital study, we present population norms for a wide range of HRQoL instruments and dimensions, some of which have not been previously published. This detailed insight into the health status of the general population in these eight countries may be valuable for health care policy and resource allocation. Moreover, data from this study can serve as reference point or baseline for future studies. Lastly, significant differences were found between countries and subgroups, highlighting the need for country-specific population norms.

## Electronic supplementary material

Below is the link to the electronic supplementary material.

Supplementary Material 1

## Data Availability

Anonymized, aggregated study data is available upon reasonable request through the corresponding author.
