# Supplementary material for: POPUP: an observational digital study reporting general population norms for the EQ-5D-5-L and HUI-3 in 8 countries
Source: Arch Public Health. 2025 Jul 11;83:184. doi: 10.1186/s13690-025-01642-z (PMC12247287; doi:10.1186/s13690-025-01642-z)
Supplement: Supplementary file 1 — Supplementary Material 1 [file 13690_2025_1642_MOESM1_ESM.docx]

**Online supp lemental Material**

**Table S1.** Prevalence of self-reported health conditions in the general population; POPUP study, January–March 2021

| **Health conditions** | **Belgium** | **Canada** | **Germany** | **Italy** | **Netherlands** | **Spain** | **UK** | **US** |
| --- | --- | --- | --- | --- | --- | --- | --- | --- |
|  | N=1000 | N=1000 | N=1000 | N=1000 | N=1000 | N=1000 | N=1000 | N=2000 |
| Diabetes with (out) complications | 6.7% | 9.6% | 9.0% | 6.1% | 9.0% | 5.9% | 7.8% | 19.3% |
| Respiratory disorder | 8.5% | 7.9% | 9.7% | 5.0% | 12.4% | 6.7% | 12.1% | 7.9% |
| Thyroid problems/disorder | 9.3% | 9.1% | 18.7% | 12.3% | 5.2% | 8.8% | 5.8% | 8.3% |
| Cardiovascular disease | 5.4% | 2.8% | 6.7% | 4.1% | 7.5% | 4.4% | 1.8% | 4.0% |
| Congestive heart failure | 0.2% | 0.9% | 1.6% | 0.6% | 0.2% | 0.6% | 0.2% | 1.9% |
| Osteoporosis | 3.6% | 2.9% | 1.6% | 6.7% | 2.3% | 2.0% | 2.7% | 3.4% |
| Rheumatoid arthritis/Psoriasis (Arthritis) | 10.7% | 7.6% | 12.7% | 8.0% | 8.0% | 7.9% | 8.2% | 6.5% |
| Crohn's disease/Ulcerative colitis | 1.8% | 1.0% | 1.5% | 1.7% | 0.8% | 1.1% | 1.7% | 2.1% |
| Systemic Lupus Erythematosus | 0.1% | 0.1% | 0.1% | 0.3% | 0.1% | 0.0% | 0.7% | 0.6% |
| Lupus nephritis | 0.0% | 0.4% | 0.0% | 0.0% | 0.2% | 0.0% | 0.0% | 0.4% |
| Multiple sclerosis | 0.4% | 0.5% | 0.5% | 0.6% | 1.0% | 0.6% | 0.5% | 0.3% |
| Gastrointestinal problems | 9.7% | 5.9% | 3.4% | 10.2% | 6.3% | 5.6% | 8.1% | 5.7% |
| Depression | 9.1% | 15.8% | 14.4% | 6.7% | 7.9% | 10.4% | 17.0% | 10.7% |
| Anxiety | 12.8% | 24.3% | 9.2% | 14.9% | 5.9% | 17.1% | 20.6% | 12.4% |
| Liver disease | 0.6% | 1.1% | 0.7% | 0.8% | 0.3% | 1.7% | 0.7% | 0.6% |
| Kidney disease | 1.4% | 0.5% | 0.7% | 0.4% | 1.1% | 0.8% | 1.0% | 1.6% |
| HIV | 0.3% | 0.3% | 0.2% | 0.0% | 0.4% | 1.1% | 0.0% | 0.5% |
| Peptic ulcer | 1.0% | 0.5% | 1.0% | 0.1% | 0.3% | 0.1% | 0.4% | 0.6% |
| Hemiplegia | 0.1% | 0.1% | 0.2% | 0.0% | 0.0% | 0.4% | 0.0% | 0.8% |
| Dementia | 0.1% | 0.3% | 0.0% | 0.0% | 0.0% | 0.0% | 0.0% | 0.1% |
| Cancer | 2.8% | 1.9% | 3.1% | 1.1% | 2.2% | 1.3% | 1.1% | 2.2% |
| No health conditions | 41.1% | 38.1% | 37.5% | 37.9% | 48.7% | 42.0% | 44.5% | 39.6% |

**Table S2.** EQ-5D-5L utility values by respondent subgroup; POPUP study, January–March 2021

| **Subgroups** | **Belgium** | **Canada** | **Germany** | **Italy** | **Netherlands** | **Spain** | **UK** | **US** | **All** |
| --- | --- | --- | --- | --- | --- | --- | --- | --- | --- |
|  | N=1000 | N=1000 | N=1000 | N=1000 | N=1000 | N=1000 | N=1000 | N=2000 | N=9000 |
| **Living situation** | **Mean, SD**  **(Q1 ; Q3), N** | **Mean, SD**  **(Q1 ; Q3), N** | **Mean, SD**  **(Q1 ; Q3), N** | **Mean, SD**  **(Q1 ; Q3), N** | **Mean, SD**  **(Q1 ; Q3), N** | **Mean, SD**  **(Q1 ; Q3), N** | **Mean, SD**  **(Q1 ; Q3), N** | **Mean, SD**  **(Q1 ; Q3), N** | **Mean, SD**  **(Q1 ; Q3), N** |
| At home with help from a caregiver | 0.330, 0.344 (0.101 ; 0.721), 11 | 0.538, 0.276 (0.339 ; 0.774), 17 | 0.561, 0.395 (0.522 ; 0.835), 21 | 0.739, 0.167 (0.632 ; 0.842), 14 | 0.515, 0.314 (0.300 ; 0.748), 39 | 0.426, 0.404 (0.435 ; 0.544), 14 | 0.316, 0.365 (0.036 ; 0.567), 33 | 0.606, 0.51 (0.317 ; 1), 70 | 0.534, 0.414 (0.304 ; 0.791), 219 |
| At home without help from a caregiver | 0.873, 0.133 (0.818 ; 1), 800 | 0.851, 0.149 (0.794 ; 1), 692 | 0.868, 0.177 (0.855 ; 1), 759 | 0.915, 0.094 (0.891 ; 1), 988 | 0.861, 0.174 (0.813 ; 1), 909 | 0.890, 0.136 (0.841 ; 1), 791 | 0.811, 0.211 (0.735 ; 1), 581 | 0.858, 0.238 (0.844 ; 1), 1297 | 0.867, 0.174 (0.818 ; 1), 6817 |
| In a long-term care rehabilitation facility | 0.394, 0.000 (0.394 ; 0.394), 1 | 0.313, 0.000 (0.313 ; 0.313), 1 | . | 0.488, 0.000 (0.488 ; 0.488), 1 | . | . | 0.452, 0.000 (0.452 ; 0.452), 1 | 0.508, 0.613 (-0.375 ; 1), 3 | 0.449, 0.359 (0.394 ; 0.452), 7 |
| In a nursing home | 0.625, 0.000 (0.625 ; 0.625), 1 | -0.163, 0.000 (-0.163 ; -0.163), 1 | -0.322, 0.441 (-0.661 ; 0.277), 2 | 0.602, 0.226 (0.537 ; 0.762), 2 | . | 0.363, 0.000 (0.363 ; 0.363), 1 | 0.399, 0.000 (0.399 ; 0.399), 1 | 0.683, 0.312 (0.609 ; 1), 10 | 0.534, 0.409 (0.385 ; 0.657), 18 |
| With a family member | 0.809, 0.193 (0.742 ; 1), 258 | 0.812, 0.183 (0.724 ; 0.922), 354 | 0.870, 0.162 (0.819 ; 0.970), 244 | 0.866, 0.153 (0.808 ; 1), 378 | 0.744, 0.208 (0.642 ; 0.879), 43 | 0.812, 0.199 (0.750 ; 0.922), 188 | 0.767, 0.247 (0.703 ; 0.879), 352 | 0.827, 0.240 (0.750 ; 1), 555 | 0.821, 0.207 (0.750 ; 1), 2372 |
| **Education** |  |  |  |  |  |  |  |  |  |
| Primary education | 0.877, 0.124 (0.815 ; 1), 36 | 0.76, 0.256 (0.643 ; 0.919), 12 | 0.855, 0.208 (0.800 ; 1), 40 | 0.909, 0.217 (0.868 ; 1), 27 | 0.805, 0.263 (0.782 ; 1), 14 | 0.836, 0.259 (0.771 ; 1), 58 | 0.634, 0.642 (0.092 ; 1), 7 | 0.556, 1.027 (0.260 ; 1), 21 | 0.781, 0.429 (0.713 ; 1), 215 |
| Secondary education | 0.841, 0.168 (0.766 ; 1), 581 | 0.813, 0.260 (0.738 ; 0.922), 263 | 0.847, 0.215 (0.813 ; 0.970), 623 | 0.879, 0.270 (0.844 ; 1), 176 | 0.831, 0.226 (0.778 ; 1), 501 | 0.863, 0.242 (0.838 ; 1), 343 | 0.772, 0.347 (0.683 ; 1), 322 | 0.849, 0.353 (0.777 ; 1), 396 | 0.837, 0.258 (0.771 ; 1), 3205 |
| Higher education | 0.868, 0.156 (0.818 ; 1), 475 | 0.841, 0.145 (0.791 ; 1), 844 | 0.898, 0.129 (0.887 ; 1), 389 | 0.903, 0.091 (0.868 ; 1), 1213 | 0.858, 0.156 (0.808 ; 1), 492 | 0.880, 0.096 (0.838 ; 1), 614 | 0.792, 0.169 (0.735 ; 1), 687 | 0.844, 0.189 (0.817 ; 1), 1598 | 0.858, 0.151 (0.815 ; 1), 6312 |
| **Employment status** |  |  |  |  |  |  |  |  |  |
| At home | 0.819, 0.157 (0.766 ; 0.922), 50 | 0.817, 0.176 (0.73 ; 0.922), 66 | 0.814, 0.199 (0.783 ; 0.97), 41 | 0.847, 0.291 (0.844 ; 1), 84 | 0.769, 0.263 (0.669 ; 1), 73 | 0.875, 0.193 (0.841 ; 1), 30 | 0.753, 0.316 (0.654 ; 1), 74 | 0.833, 0.240 (0.711 ; 1), 111 | 0.817, 0.248 (0.751 ; 1), 529 |
| Employed | 0.889, 0.140 (0.841 ; 1), 512 | 0.848, 0.154 (0.794 ; 1), 633 | 0.897, 0.141 (0.881 ; 1), 721 | 0.912, 0.108 (0.902 ; 1), 701 | 0.875, 0.173 (0.817 ; 1), 454 | 0.900, 0.114 (0.841 ; 1), 579 | 0.823, 0.195 (0.750 ; 1), 599 | 0.837, 0.291 (0.817 ; 1), 985 | 0.869, 0.186 (0.822 ; 1), 5184 |
| On sick leave | 0.603, 0.258 (0.494 ; 0.774), 65 | 0.574, 0.240 (0.480 ; 0.690), 18 | 0.561, 0.379 (0.509 ; 0.720), 19 | 0.727, 0.088 (0.579 ; 0.852), 3 | 0.642, 0.255 (0.459 ; 0.844), 53 | 0.521, 0.234 (0.351 ; 0.757), 15 | 0.316, 0.318 (0.119 ; 0.533), 30 | 0.654, 0.258 (0.501 ; 0.877), 27 | 0.569, 0.291 (0.423 ; 0.766), 230 |
| Retired | 0.852, 0.122 (0.794 ; 0.922), 386 | 0.813, 0.186 (0.756 ; 0.922), 263 | 0.778, 0.350 (0.663 ; 0.943), 86 | 0.875, 0.125 (0.818 ; 0.956), 360 | 0.850, 0.162 (0.804 ; 1), 326 | 0.725, 0.416 (0.550 ; 0.922), 49 | 0.783, 0.190 (0.683 ; 1), 160 | 0.854, 0.139 (0.777 ; 1), 713 | 0.833, 0.173 (0.776 ; 1), 2343 |
| Student | 0.891, 0.125 (0.794 ; 1), 37 | 0.834, 0.212 (0.652 ; 1), 49 | 0.904, 0.098 (0.877 ; 1), 138 | 0.917, 0.068 (0.891 ; 0.956), 138 | 0.868, 0.182 (0.809 ; 1), 46 | 0.894, 0.084 (0.841 ; 1), 201 | 0.759, 0.310 (0.635 ; 1), 45 | 0.730, 0.686 (0.449 ; 1), 20 | 0.854, 0.186 (0.809 ; 1), 674 |
| Unemployed | 0.814, 0.170 (0.727 ; 0.922), 39 | 0.787, 0.252 (0.73 ; 0.922), 90 | 0.789, 0.223 (0.783 ; 0.917), 47 | 0.889, 0.132 (0.852 ; 1), 128 | 0.788, 0.245 (0.717 ; 1), 53 | 0.823, 0.212 (0.730 ; 1), 137 | 0.716, 0.302 (0.567 ; 0.879), 105 | 0.837, 0.344 (0.817 ; 1), 156 | 0.811, 0.259 (0.736 ; 1), 755 |
| **Health conditions** |  |  |  |  |  |  |  |  |  |
| Diabetes with(out) chronic complications | 0.795, 0.145 (0.666 ; 0.922), 84 | 0.748, 0.231 (0.620 ; 0.922), 109 | 0.696, 0.342 (0.518 ; 0.917), 69 | 0.828, 0.164 (0.808 ; 0.953), 95 | 0.797, 0.190 (0.717 ; 1), 99 | 0.818, 0.217 (0.771 ; 0.922), 51 | 0.652, 0.285 (0.516 ; 0.877), 80 | 0.715, 0.309 (0.636 ; 0.943), 399 | 0.740, 0.268 (0.643 ; 0.940), 986 |
| Respiratory disease (e.g., asthma, COPD) | 0.744, 0.212 (0.675 ; 0.899), 95 | 0.715, 0.192 (0.605 ; 0.872), 85 | 0.700, 0.299 (0.506 ; 0.917), 96 | 0.839, 0.146 (0.795 ; 0.953), 80 | 0.735, 0.245 (0.705 ; 0.887), 129 | 0.778, 0.258 (0.690 ; 0.922), 78 | 0.691, 0.259 (0.627 ; 0.877), 123 | 0.702, 0.354 (0.513 ; 1), 156 | 0.726, 0.265 (0.635 ; 0.913), 842 |
| Thyroid problems or disorder | 0.808, 0.200 (0.771 ; 0.922), 95 | 0.747, 0.197 (0.637 ; 0.922), 86 | 0.834, 0.248 (0.819 ; 0.943), 129 | 0.771, 0.260 (0.635 ; 0.953), 123 | 0.751, 0.229 (0.705 ; 0.887), 46 | 0.851, 0.148 (0.794 ; 0.922), 68 | 0.593, 0.315 (0.480 ; 0.879), 58 | 0.680, 0.295 (0.501 ; 0.940), 177 | 0.763, 0.258 (0.674 ; 0.943), 782 |
| Cardiovascular disease | 0.763, 0.213 (0.722 ; 0.919), 71 | 0.691, 0.198 (0.561 ; 0.919), 34 | 0.727, 0.326 (0.663 ; 0.917), 52 | 0.734, 0.211 (0.721 ; 0.949), 72 | 0.754, 0.203 (0.679 ; 0.887), 86 | 0.741, 0.355 (0.713 ; 0.899), 32 | 0.431, 0.336 (0.127 ; 0.693), 21 | 0.597, 0.369 (0.229 ; 0.902), 82 | 0.699, 0.285 (0.617 ; 0.902), 450 |
| Congestive heart failure | 0.081, 0.341 (-0.108 ; 0.456), 2 | 0.622, 0.293 (0.322 ; 0.919), 9 | 0.747, 0.238 (0.518 ; 0.917), 12 | 0.693, 0.180 (0.488 ; 0.953), 10 | 0.780, 0.233 (0.489 ; 1), 3 | 0.731, 0.285 (0.771 ; 0.838), 4 | 0.295, 0.357 (-0.328 ; 0.631), 5 | 0.505, 0.358 (0.133 ; 0.810), 42 | 0.591, 0.331 (0.363 ; 0.883), 87 |
| Osteoporosis | 0.683, 0.241 (0.539 ; 0.873), 48 | 0.725, 0.189 (0.556 ; 0.899), 39 | 0.615, 0.359 (0.391 ; 0.891), 15 | 0.815, 0.182 (0.767 ; 0.953), 68 | 0.653, 0.302 (0.618 ; 0.813), 23 | 0.476, 0.502 (0.351 ; 0.818), 12 | 0.420, 0.367 (0.036 ; 0.671), 28 | 0.752, 0.217 (0.615 ; 0.940), 85 | 0.689, 0.280 (0.544 ; 0.909), 318 |
| Rheumatoid arthritis | 0.678, 0.247 (0.541 ; 0.878), 75 | 0.660, 0.178 (0.507 ; 0.841), 42 | 0.691, 0.307 (0.505 ; 0.865), 53 | 0.722, 0.192 (0.619 ; 0.902), 50 | 0.670, 0.212 (0.523 ; 0.83), 55 | 0.610, 0.234 (0.518 ; 0.794), 33 | 0.580, 0.272 (0.533 ; 0.725), 50 | 0.674, 0.244 (0.535 ; 0.883), 75 | 0.662, 0.243 (0.533 ; 0.849), 433 |
| Crohn’s disease | 0.696, 0.240 (0.525 ; 0.878), 8 | 0.878, 0.095 (0.919 ; 0.922), 6 | 0.798, 0.220 (0.835 ; 0.913), 9 | 0.764, 0.114 (0.697 ; 0.852), 5 | 0.505, 0.593 (-0.158 ; 0.848), 3 | 0.697, 0.139 (0.576 ; 0.841), 4 | 0.488, 0.384 (0.221 ; 0.735), 10 | 0.624, 0.370 (0.474 ; 0.940), 16 | 0.662, 0.312 (0.501 ; 0.883), 61 |
| Ulcerative colitis | 0.672, 0.310 (0.584 ; 0.899), 6 | 0.867, 0.082 (0.841 ; 0.922), 8 | 0.692, 0.358 (0.322 ; 0.943), 6 | 0.741, 0.283 (0.488 ; 1), 11 | 0.742, 0.238 (0.782 ; 0.848), 5 | 0.823, 0.145 (0.663 ; 0.878), 8 | 0.498, 0.433 (-0.218 ; 0.837), 10 | 0.767, 0.437 (0.501 ; 1), 17 | 0.732, 0.333 (0.584 ; 1), 71 |
| Systemic lupus erythematosus | 0.698, 0.250 (0.476 ; 0.899), 2 | 0.792, 0.020 (0.771 ; 0.805), 2 | 0.748, 0.322 (0.277 ; 0.913), 2 | 0.564, 0.312 (0.230 ; 0.839), 6 | 0.848, 0.000 (0.848 ; 0.848), 1 | 0.637, 0.000 (0.637 ; 0.637), 1 | 0.222, 0.496 (-0.218 ; 0.671), 5 | 0.486, 0.418 (0.321 ; 0.902), 10 | 0.472, 0.383 (0.321 ; 0.805), 29 |
| Lupus nephritis | . | 0.933, 0.088 (0.922 ; 1), 3 | 0.277, 0.000 (0.277 ; 0.277), 1 | . | 0.777, 0.007 (0.773 ; 0.782), 2 | **.** | **.** | 0.457, 0.332 (0.229 ; 0.501), 9 | 0.618, 0.334 (0.501 ; 0.922), 15 |
| Psoriasis | 0.796, 0.249 (0.713 ; 0.922), 43 | 0.760, 0.178 (0.616 ; 0.919), 39 | 0.791, 0.232 (0.756 ; 0.943), 42 | 0.859, 0.151 (0.844 ; 0.956), 54 | 0.685, 0.303 (0.642 ; 0.883), 28 | 0.866, 0.126 (0.818 ; 1), 37 | 0.622, 0.257 (0.516 ; 0.794), 36 | 0.670, 0.235 (0.501 ; 0.940), 45 | 0.762, 0.228 (0.664 ; 0.922), 324 |
| Psoriatic arthritis | 0.525, 0.139 (0.405 ; 0.573), 4 | 0.697, 0.153 (0.574 ; 0.794), 13 | 0.729, 0.275 (0.506 ; 0.887), 14 | 0.852, 0.081 (0.765 ; 0.909), 7 | 0.620, 0.348 (0.270 ; 0.861), 4 | 0.543, 0.229 (0.286 ; 0.729), 7 | 0.538, 0.257 (0.304 ; 0.710), 7 | 0.539, 0.239 (0.295 ; 0.703), 24 | 0.630, 0.240 (0.480 ; 0.800), 80 |
| Multiple sclerosis | 0.517, 0.350 (0.101 ; 0.828), 5 | 0.688, 0.427 (0.149 ; 0.922), 4 | 0.713, 0.174 (0.678 ; 0.770), 6 | 0.690, 0.327 (0.449 ; 0.953), 8 | 0.618, 0.338 (0.634 ; 0.773), 9 | 0.844, 0.261 (0.878 ; 0.916), 4 | 0.218, 0.281 (0.036 ; 0.304), 5 | 0.566, 0.149 (0.501 ; 0.687), 9 | 0.615, 0.312 (0.449 ; 0.878), 50 |
| Gastrointestinal problems | 0.717, 0.237 (0.615 ; 0.872), 107 | 0.727, 0.175 (0.662 ; 0.855), 66 | 0.783, 0.240 (0.799 ; 0.917), 38 | 0.827, 0.151 (0.793 ; 0.953), 146 | 0.742, 0.209 (0.594 ; 0.883), 63 | 0.731, 0.183 (0.663 ; 0.872), 60 | 0.558, 0.295 (0.437 ; 0.767), 90 | 0.647, 0.335 (0.501 ; 0.940), 117 | 0.710, 0.250 (0.592 ; 0.891), 687 |
| Depression | 0.641, 0.249 (0.551 ; 0.794), 84 | 0.678, 0.195 (0.574 ; 0.841), 168 | 0.646, 0.267 (0.509 ; 0.861), 160 | 0.742, 0.164 (0.635 ; 0.858), 89 | 0.588, 0.241 (0.466 ; 0.742), 76 | 0.638, 0.257 (0.519 ; 0.794), 101 | 0.541, 0.317 (0.321 ; 0.767), 173 | 0.632, 0.331 (0.501 ; 0.877), 203 | 0.631, 0.273 (0.508 ; 0.841), 1054 |
| Anxiety | 0.682, 0.222 (0.608 ; 0.841), 132 | 0.726, 0.172 (0.643 ; 0.841), 250 | 0.662, 0.253 (0.506 ; 0.877), 104 | 0.806, 0.135 (0.753 ; 0.909), 223 | 0.591, 0.238 (0.492 ; 0.791), 53 | 0.721, 0.220 (0.627 ; 0.841), 183 | 0.589, 0.306 (0.508 ; 0.768), 200 | 0.666, 0.323 (0.535 ; 0.883), 225 | 0.686, 0.247 (0.585 ; 0.872), 1370 |
| Liver disease | 0.644, 0.174 (0.500 ; 0.828), 7 | 0.733, 0.180 (0.628 ; 0.873), 13 | 0.612, 0.281 (0.391 ; 0.907), 8 | 0.724, 0.181 (0.619 ; 0.953), 18 | 0.676, 0.228 (0.379 ; 0.813), 4 | 0.666, 0.569 (0.655 ; 0.922), 11 | 0.578, 0.315 (0.426 ; 0.837), 14 | 0.607, 0.256 (0.501 ; 0.877), 19 | 0.657, 0.288 (0.501 ; 0.907), 94 |
| Kidney disease | 0.830, 0.134 (0.766 ; 0.922), 17 | 0.608, 0.322 (0.333 ; 0.841), 6 | 0.763, 0.226 (0.799 ; 0.913), 9 | 0.580, 0.311 (0.249 ; 0.709), 4 | 0.537, 0.363 (0.199 ; 0.848), 11 | 0.813, 0.100 (0.755 ; 0.916), 6 | 0.579, 0.349 (0.466 ; 0.837), 14 | 0.623, 0.322 (0.474 ; 0.94), 39 | 0.664, 0.299 (0.501 ; 0.916), 106 |
| HIV | 0.750, 0.230 (0.54 ; 1), 3 | 0.620, 0.180 (0.500 ; 0.620), 3 | 0.840, 0.170 (0.880 ; 1), 3 | 1, 0.000 (1 ; 1), 1 | 0.810, 0.130 (0.71 ; 0.89), 4 | 0.700, 0.200 (0.480 ; 0.82), 10 | 1, . (1 ; 1), 1 | 0.680, 0.360 (0.5000 ; 1), 10 | 0.720, 0.240 (0.500 ; 0.940), 35 |
| Peptic ulcer disease | 0.736, 0.235 (0.744 ; 0.841), 12 | 0.727, 0.146 (0.674 ; 0.818), 5 | 0.801, 0.205 (0.785 ; 0.913), 13 | 0.249, 0.000 (0.249 ; 0.249), 1 | 0.724, 0.175 (0.808 ; 0.822), 4 | 0.870, 0.132 (0.746 ; 1), 2 | 0.806, 0.232 (0.516 ; 1), 5 | 0.324, 1.177 (-0.551 ; 0.940), 4 | 0.646, 0.411 (0.501 ; 0.907), 46 |
| Hemiplegia/paraplegia | 0.916, 0.000 (0.916 ; 0.916), 1 | 0.805, 0.000 (0.805 ; 0.805), 1 | 0.609, 0.158 (0.669 ; 0.734), 3 | 0.129, 0.000 (0.129 ; 0.129), 1 | . | -0.149, 0.665 (-0.416 ; 0.316), 2 | 0.686, 0.000 (0.686 ; 0.686), 1 | 0.542, 0.379 (0.501 ; 0.712), 7 | 0.458, 0.458 (0.317 ; 0.712), 16 |
| Dementia | 0.794, 0.000 (0.794 ; 0.794), 1 | 0.449, 0.141 (0.408 ; 0.620), 3 | . | . | 1, . (1 ; 1), 1 | . | . | 0.140, 0.430 (0.012 ; 0.012), 3 | 0.433, 0.351 (0.012 ; 0.794), 8 |
| Cancer | 0.827, 0.120 (0.722 ; 0.922), 34 | 0.718, 0.258 (0.666 ; 0.922), 24 | 0.708, 0.338 (0.588 ; 0.943), 18 | 0.863, 0.074 (0.803 ; 0.953), 18 | 0.829, 0.126 (0.787 ; 0.887), 26 | 0.678, 0.410 (0.351 ; 0.922), 7 | 0.599, 0.212 (0.592 ; 0.739), 15 | 0.719, 0.183 (0.660 ; 0.845), 57 | 0.746, 0.211 (0.660 ; 0.919), 199 |
| None of the above | 0.925, 0.103 (0.873 ; 1), 406 | 0.911, 0.144 (0.872 ; 1), 423 | 0.943, 0.098 (0.917 ; 1), 467 | 0.949, 0.076 (0.953 ; 1), 519 | 0.915, 0.126 (0.883 ; 1), 467 | 0.936, 0.103 (0.919 ; 1), 449 | 0.888, 0.159 (0.837 ; 1), 441 | 0.940, 0.153 (0.940 ; 1), 673 | 0.927, 0.126 (0.887 ; 1), 3845 |

**Table S3.** HUI-3 utility values by respondent subgroup; POPUP study, January–March 2021

| **Subgroups** | **Belgium** | **Canada** | **Germany** | **Italy** | **Netherlands** | **Spain** | **UK** | **US** | **All** |
| --- | --- | --- | --- | --- | --- | --- | --- | --- | --- |
|  | N=1000 | N=1000 | N=1000 | N=1000 | N=1000 | N=1000 | N=1000 | N=2000 | N=9000 |
| **Living situation** | **Mean, SD**  **(Q1 ; Q3), N** | **Mean, SD**  **(Q1 ; Q3), N** | **Mean, SD**  **(Q1 ; Q3), N** | **Mean, SD**  **(Q1 ; Q3), N** | **Mean, SD**  **(Q1 ; Q3), N** | **Mean, SD (Q1 ; Q3), N** | **Mean, SD**  **(Q1 ; Q3), N** | **Mean, SD**  **(Q1 ; Q3), N** | **Mean, SD**  **(Q1 ; Q3), N** |
| At home with help from a caregiver | 0.230, 0.358 (-0.26 ; 0.61), 11 | 0.304, 0.321 (0.07 ; 0.57), 17 | 0.249, 0.354 (-0.10 ; 0.69), 21 | 0.374, 0.400 (-0.21 ; 0.67), 14 | 0.326, 0.386 (-0.03 ; 0.62), 39 | 0.195, 0.315 (0.07 ; 0.36), 14 | 0.071, 0.279 (-0.18 ; 0.26), 33 | 0.359, 0.427 (0.16 ; 0.59), 70 | 0.293, 0.384 (0.04 ; 0.57), 219 |
| At home without help from a caregiver | 0.782, 0.216 (0.70 ; 0.93), 800 | 0.781, 0.234 (0.71 ; 0.97), 692 | 0.761, 0.244 (0.67 ; 0.92), 759 | 0.807, 0.165 (0.74 ; 0.93), 988 | 0.806, 0.223 (0.73 ; 0.97), 909 | 0.791, 0.230 (0.73 ; 0.93), 791 | 0.744, 0.275 (0.65 ; 0.93), 581 | 0.792, 0.267 (0.73 ; 0.97), 1297 | 0.785, 0.234 (0.72 ; 0.95), 6817 |
| In a long-term care rehabilitation facility | -0.300, 0.000 (-0.30 ; -0.30), 1 | -0.280, 0.000 (-0.28 ; -0.28), 1 | . | 0.550, 0.000 (0.55 ; 0.55), 1 | . | . | 1, 0.000 (1 ; 1), 1 | 0.301, 0.465 (-0.10 ; 0.97), 3 | 0.504, 0.590 (-0.1 ; 1), 7 |
| In a nursing home | -0.030, 0.000 (-0.03 ; -0.03), 1 | -0.350, 0.000 (-0.35 ; -0.35), 1 | -0.285, 0.085 (-0.35 ; -0.17), 2 | -0.097, 0.06 (-0.14 ; -0.08), 2 | . | -0.200, 0.000 (-0.2 ; -0.2), 1 | -0.130, 0.000 (-0.13 ; -0.13), 1 | 0.437, 0.519 (-0.01 ; 1), 10 | 0.131, 0.514 (-0.13 ; 0.28), 18 |
| With a family member | 0.693, 0.266 (0.54 ; 0.92), 258 | 0.692, 0.289 (0.54 ; 0.92), 354 | 0.744, 0.251 (0.62 ; 0.92), 244 | 0.744, 0.237 (0.68 ; 0.91), 378 | 0.627, 0.342 (0.38 ; 0.91), 43 | 0.684, 0.304 (0.56 ; 0.91), 188 | 0.705, 0.297 (0.63 ; 0.92), 352 | 0.687, 0.356 (0.55 ; 0.97), 555 | 0.703, 0.297 (0.59 ; 0.92), 2372 |
| **Education** |  |  |  |  |  |  |  |  |  |
| Primary education | 0.762, 0.236 (0.63 ; 0.97), 36 | 0.612, 0.475 (0.31 ; 0.88), 12 | 0.747, 0.293 (0.61 ; 0.92), 40 | 0.854, 0.172 (0.77 ; 0.93), 27 | 0.783, 0.260 (0.71 ; 0.97), 14 | 0.682, 0.468 (0.54 ; 0.91), 58 | 0.366, 0.960 (-0.27 ; 1), 7 | 0.485, 0.906 (-0.01 ; 0.90), 21 | 0.671, 0.492 (0.56 ; 0.92), 215 |
| Secondary education | 0.734, 0.258 (0.63 ; 0.92), 581 | 0.712, 0.384 (0.56 ; 0.93), 263 | 0.729, 0.283 (0.65 ; 0.92), 623 | 0.750, 0.438 (0.71 ; 0.92), 176 | 0.767, 0.288 (0.67 ; 0.97), 501 | 0.757, 0.358 (0.68 ; 0.93), 343 | 0.690, 0.428 (0.56 ; 0.91), 322 | 0.703, 0.569 (0.55 ; 0.97), 396 | 0.727, 0.369 (0.62 ; 0.93), 3205 |
| Higher education | 0.782, 0.221 (0.71 ; 0.93), 475 | 0.760, 0.220 (0.67 ; 0.97), 844 | 0.777, 0.221 (0.71 ; 0.95), 389 | 0.792, 0.142 (0.73 ; 0.93), 1213 | 0.799, 0.221 (0.71 ; 0.97), 492 | 0.790, 0.148 (0.73 ; 0.93), 614 | 0.751, 0.203 (0.67 ; 0.93), 687 | 0.797, 0.188 (0.74 ; 0.97), 1598 | 0.782, 0.191 (0.71 ; 0.97), 6312 |
| **Employment status** |  |  |  |  |  |  |  |  |  |
| At home | 0.780, 0.216 (0.71 ; 0.92), 50 | 0.721, 0.289 (0.60 ; 0.95), 66 | 0.673, 0.248 (0.54 ; 0.85), 41 | 0.765, 0.319 (0.73 ; 0.91), 84 | 0.716, 0.320 (0.63 ; 0.92), 73 | 0.784, 0.236 (0.73 ; 0.91), 30 | 0.670, 0.353 (0.43 ; 0.91), 74 | 0.712, 0.391 (0.60 ; 0.97), 111 | 0.726, 0.321 (0.63 ; 0.92), 529 |
| Employed | 0.786, 0.245 (0.73 ; 0.93), 512 | 0.749, 0.269 (0.62 ; 0.97), 633 | 0.779, 0.234 (0.72 ; 0.93), 721 | 0.789, 0.187 (0.73 ; 0.93), 701 | 0.821, 0.253 (0.77 ; 0.97), 454 | 0.811, 0.212 (0.77 ; 0.93), 579 | 0.775, 0.244 (0.71 ; 0.93), 599 | 0.744, 0.332 (0.63 ; 0.97), 985 | 0.777, 0.257 (0.71 ; 0.97), 5184 |
| On sick leave | 0.434, 0.307 (0.27 ; 0.67), 65 | 0.503, 0.276 (0.31 ; 0.73), 18 | 0.288, 0.367 (0.06 ; 0.47), 19 | 0.303, 0.158 (0.15 ; 0.60), 3 | 0.555, 0.307 (0.34 ; 0.79), 53 | 0.292, 0.352 (-0.11 ; 0.6), 15 | 0.197, 0.288 (0.01 ; 0.49), 30 | 0.515, 0.435 (0.14 ; 0.92), 27 | 0.420, 0.346 (0.18 ; 0.67), 230 |
| Retired | 0.759, 0.186 (0.66 ; 0.92), 386 | 0.744, 0.225 (0.65 ; 0.92), 263 | 0.675, 0.394 (0.54 ; 0.91), 86 | 0.758, 0.212 (0.72 ; 0.91), 360 | 0.777, 0.192 (0.64 ; 0.95), 326 | 0.616, 0.446 (0.43 ; 0.92), 49 | 0.704, 0.265 (0.61 ; 0.91), 160 | 0.782, 0.178 (0.70 ; 0.95), 713 | 0.748, 0.221 (0.65 ; 0.92), 2343 |
| Student | 0.794, 0.240 (0.71 ; 0.92), 37 | 0.735, 0.369 (0.67 ; 0.93), 49 | 0.793, 0.183 (0.71 ; 0.93), 138 | 0.773, 0.181 (0.68 ; 0.93), 138 | 0.863, 0.142 (0.78 ; 0.97), 46 | 0.774, 0.186 (0.67 ; 0.93), 201 | 0.661, 0.467 (0.54 ; 0.95), 45 | 0.436, 0.887 (-0.09 ; 0.93), 20 | 0.730, 0.295 (0.66 ; 0.93), 674 |
| Unemployed | 0.742, 0.226 (0.58 ; 0.92), 39 | 0.694, 0.323 (0.60 ; 0.92), 90 | 0.705, 0.254 (0.54 ; 0.88), 47 | 0.799, 0.215 (0.73 ; 0.93), 128 | 0.693, 0.341 (0.57 ; 0.95), 53 | 0.669, 0.354 (0.45 ; 0.92), 137 | 0.604, 0.383 (0.41 ; 0.88), 105 | 0.701, 0.465 (0.57 ; 0.97), 156 | 0.695, 0.354 (0.56 ; 0.93), 755 |
| **Health conditions** |  |  |  |  |  |  |  |  |  |
| Diabetes with(out) chronic complications | 0.666, 0.244 (0.50 ; 0.85), 84 | 0.672, 0.309 (0.51 ; 0.93), 109 | 0.579, 0.356 (0.45 ; 0.85), 69 | 0.748, 0.160 (0.66 ; 0.85), 95 | 0.742, 0.217 (0.58 ; 0.92), 99 | 0.666, 0.355 (0.48 ; 0.91), 51 | 0.548, 0.353 (0.42 ; 0.85), 80 | 0.627, 0.333 (0.37 ; 0.93), 399 | 0.645, 0.309 (0.47 ; 0.91), 986 |
| Respiratory disease (e.g., asthma, COPD) | 0.623, 0.302 (0.49 ; 0.85), 95 | 0.536, 0.327 (0.35 ; 0.79), 85 | 0.588, 0.335 (0.44 ; 0.85), 96 | 0.681, 0.212 (0.54 ; 0.91), 80 | 0.623, 0.290 (0.45 ; 0.85), 129 | 0.612, 0.330 (0.34 ; 0.91), 78 | 0.576, 0.329 (0.42 ; 0.85), 123 | 0.581, 0.347 (0.35 ; 0.91), 156 | 0.597, 0.315 (0.37 ; 0.85), 842 |
| Thyroid problems or disorder | 0.757, 0.221 (0.69 ; 0.91), 95 | 0.599, 0.349 (0.31 ; 0.92), 86 | 0.702, 0.300 (0.57 ; 0.88), 129 | 0.628, 0.324 (0.40 ; 0.91), 123 | 0.579, 0.315 (0.25 ; 0.91), 46 | 0.774, 0.206 (0.72 ; 0.91), 68 | 0.501, 0.337 (0.23 ; 0.82), 58 | 0.624, 0.295 (0.35 ; 0.92), 177 | 0.658, 0.305 (0.48 ; 0.91), 782 |
| Cardiovascular disease | 0.643, 0.260 (0.48 ; 0.85), 71 | 0.508, 0.294 (0.30 ; 0.77), 34 | 0.571, 0.390 (0.22 ; 0.85), 52 | 0.558, 0.222 (0.41 ; 0.73), 72 | 0.659, 0.266 (0.57 ; 0.89), 86 | 0.601, 0.434 (0.54 ; 0.88), 32 | 0.318, 0.365 (0.04 ; 0.73), 21 | 0.425, 0.417 (0.09 ; 0.83), 82 | 0.555, 0.338 (0.3 ; 0.85), 450 |
| Congestive heart failure | 0.214, 0.314 (0.04 ; 0.56), 2 | 0.444, 0.476 (-0.05 ; 0.91), 9 | 0.548, 0.341 (0.21 ; 0.74), 12 | 0.614, 0.255 (0.66 ; 0.91), 10 | 0.516, 0.536 (-0.17 ; 1), 3 | 0.463, 0.545 (0.43 ; 0.77), 4 | 0.204, 0.365 (-0.34 ; 0.68), 5 | 0.399, 0.400 (0.01 ; 0.82), 42 | 0.449, 0.389 (0.09 ; 0.83), 87 |
| Osteoporosis | 0.564, 0.288 (0.31 ; 0.85), 48 | 0.642, 0.243 (0.44 ; 0.88), 39 | 0.474, 0.393 (0.16 ; 0.74), 15 | 0.690, 0.281 (0.63 ; 0.91), 68 | 0.553, 0.337 (0.34 ; 0.82), 23 | 0.335, 0.609 (-0.11 ; 0.85), 12 | 0.234, 0.355 (-0.09 ; 0.42), 28 | 0.612, 0.321 (0.36 ; 0.92), 85 | 0.559, 0.345 (0.32 ; 0.85), 318 |
| Rheumatoid arthritis | 0.566, 0.302 (0.39 ; 0.85), 75 | 0.490, 0.326 (0.35 ; 0.73), 42 | 0.612, 0.372 (0.47 ; 0.84), 53 | 0.520, 0.324 (0.09 ; 0.85), 50 | 0.564, 0.263 (0.41 ; 0.78), 55 | 0.437, 0.326 (0.26 ; 0.62), 33 | 0.412, 0.353 (0.12 ; 0.79), 50 | 0.478, 0.367 (0.09 ; 0.79), 75 | 0.517, 0.335 (0.29 ; 0.79), 433 |
| Crohn’s disease | 0.595, 0.252 (0.39 ; 0.74), 8 | 0.822, 0.146 (0.76 ; 0.92), 6 | 0.631, 0.299 (0.52 ; 0.84), 9 | 0.217, 0.477 (-0.07 ; 0.73), 5 | 0.504, 0.563 (-0.10 ; 0.95), 3 | 0.301, 0.227 (0.12 ; 0.26), 4 | 0.453, 0.383 (0.26 ; 0.73), 10 | 0.498, 0.494 (0.08 ; 0.95), 16 | 0.515, 0.390 (0.25 ; 0.85), 61 |
| Ulcerative colitis | 0.532, 0.424 (0.11 ; 0.88), 6 | 0.761, 0.140 (0.66 ; 0.92), 8 | 0.492, 0.585 (-0.11 ; 0.95), 6 | 0.760, 0.185 (0.64 ; 0.97), 11 | 0.745, 0.233 (0.57 ; 0.95), 5 | 0.718, 0.227 (0.48 ; 0.88), 8 | 0.444, 0.470 (-0.31 ; 0.95), 10 | 0.737, 0.449 (0.82 ; 0.97), 17 | 0.666, 0.376 (0.53 ; 0.95), 71 |
| Systemic lupus erythematosus | 0.339, 0.437 (-0.05 ; 0.69), 2 | 0.530, 0.359 (0.30 ; 0.92), 2 | 0.460, 0.431 (-0.17 ; 0.68), 2 | 0.439, 0.291 (0.31 ; 0.83), 6 | 0.780, 0.000 (0.78 ; 0.78), 1 | -0.250, 0.000 (-0.25 ; -0.25), 1 | 0.115, 0.320 (-0.24 ; 0.26), 5 | 0.249, 0.488 (-0.07 ; 0.85), 10 | 0.268, 0.383 (-0.05 ; 0.68), 29 |
| Lupus nephritis | . | 0.687, 0.306 (0.47 ; 0.97), 3 | -0.170, 0.000 (-0.17 ; -0.17), 1 | . | 0.772, 0.247 (0.57 ; 0.91), 2 | . | . | 0.120, 0.387 (-0.07 ; 0.15), 9 | 0.351, 0.445 (-0.07 ; 0.77), 15 |
| Psoriasis | 0.691, 0.318 (0.54 ; 0.92), 43 | 0.671, 0.305 (0.46 ; 0.93), 39 | 0.639, 0.302 (0.44 ; 0.88), 42 | 0.783, 0.151 (0.73 ; 0.91), 54 | 0.554, 0.356 (0.24 ; 0.91), 28 | 0.750, 0.200 (0.68 ; 0.91), 37 | 0.582, 0.306 (0.34 ; 0.88), 36 | 0.535, 0.295 (0.32 ; 0.85), 45 | 0.656, 0.287 (0.47 ; 0.91), 324 |
| Psoriatic arthritis | 0.515, 0.369 (0.08 ; 0.70), 4 | 0.617, 0.291 (0.39 ; 0.85), 13 | 0.613, 0.345 (0.44 ; 0.85), 14 | 0.707, 0.096 (0.70 ; 0.73), 7 | 0.327, 0.418 (-0.09 ; 0.59), 4 | 0.098, 0.399 (-0.24 ; 0.37), 7 | 0.356, 0.316 (0.05 ; 0.65), 7 | 0.409, 0.425 (-0.07 ; 0.92), 24 | 0.469, 0.377 (0.05 ; 0.83), 80 |
| Multiple sclerosis | 0.367, 0.341 (0.28 ; 0.67), 5 | 0.549, 0.327 (0.23 ; 0.79), 4 | 0.469, 0.213 (0.36 ; 0.58), 6 | 0.691, 0.203 (0.43 ; 0.88), 8 | 0.637, 0.380 (0.33 ; 0.95), 9 | 0.873, 0.446 (0.93 ; 1), 4 | 0.137, 0.259 (-0.09 ; 0.19), 5 | 0.185, 0.291 (-0.07 ; 0.57), 9 | 0.510, 0.364 (0.23 ; 0.85), 50 |
| Gastrointestinal problems | 0.580, 0.310 (0.38 ; 0.85), 107 | 0.584, 0.302 (0.35 ; 0.85), 66 | 0.642, 0.277 (0.39 ; 0.88), 38 | 0.723, 0.173 (0.62 ; 0.91), 146 | 0.597, 0.288 (0.36 ; 0.79), 63 | 0.582, 0.293 (0.38 ; 0.85), 60 | 0.429, 0.320 (0.19 ; 0.71), 90 | 0.520, 0.421 (0.05 ; 0.92), 117 | 0.578, 0.314 (0.38 ; 0.85), 687 |
| Depression | 0.469, 0.348 (0.25 ; 0.71), 84 | 0.476, 0.318 (0.24 ; 0.75), 168 | 0.474, 0.282 (0.21 ; 0.7), 160 | 0.515, 0.238 (0.31 ; 0.73), 89 | 0.456, 0.347 (0.15 ; 0.75), 76 | 0.402, 0.325 (0.25 ; 0.68), 101 | 0.413, 0.348 (0.12 ; 0.69), 173 | 0.478, 0.416 (0.06 ; 0.85), 203 | 0.459, 0.339 (0.21 ; 0.74), 1054 |
| Anxiety | 0.582, 0.306 (0.44 ; 0.80), 132 | 0.590, 0.307 (0.39 ; 0.85), 250 | 0.530, 0.282 (0.31 ; 0.76), 104 | 0.648, 0.212 (0.50 ; 0.85), 223 | 0.530, 0.337 (0.34 ; 0.78), 53 | 0.535, 0.333 (0.31 ; 0.8), 183 | 0.468, 0.359 (0.22 ; 0.77), 200 | 0.558, 0.383 (0.29 ; 0.85), 225 | 0.556, 0.322 (0.34 ; 0.84), 1370 |
| Liver disease | 0.504, 0.332 (0.44 ; 0.76), 7 | 0.663, 0.222 (0.56 ; 0.88), 13 | 0.587, 0.356 (0.46 ; 0.92), 8 | 0.631, 0.179 (0.51 ; 0.85), 18 | 0.531, 0.359 (0.05 ; 0.73), 4 | 0.579, 0.536 (0.38 ; 0.85), 11 | 0.534, 0.368 (0.28 ; 0.95), 14 | 0.399, 0.312 (-0.07 ; 0.82), 19 | 0.553, 0.327 (0.36 ; 0.85), 94 |
| Kidney disease | 0.685, 0.220 (0.53 ; 0.91), 17 | 0.505, 0.222 (0.39 ; 0.56), 6 | 0.593, 0.173 (0.49 ; 0.67), 9 | 0.545, 0.188 (0.39 ; 0.54), 4 | 0.547, 0.382 (0.13 ; 0.84), 11 | 0.579, 0.386 (0.61 ; 0.85), 6 | 0.577, 0.378 (0.19 ; 0.95), 14 | 0.492, 0.412 (-0.02 ; 0.88), 39 | 0.556, 0.342 (0.35 ; 0.85), 106 |
| HIV | 0.530, 0.430 (0.08 ; 0.93), 3 | 0.300, 0.490 (-0.24 ; 0.42), 3 | 0.790, 0.180 (0.77 ; 1), 3 | 0.970, 0.000 (0.97 ; 0.97), 1 | 0.450, 0.520 (-0.11 ; 0.92), 4 | 0.440, 0.400 (0.08 ; 0.85), 10 | 1, 0.000 (1 ; 1), 1 | 0.460, 0.450 (-0.07 ; 0.85), 10 | 0.470, 0.400 (0.03 ; 0.85), 35 |
| Peptic ulcer disease | 0.619, 0.251 (0.31 ; 0.88), 12 | 0.703, 0.151 (0.67 ; 0.85), 5 | 0.683, 0.218 (0.61 ; 0.89), 13 | 0.540, 0.000 (0.54 ; 0.54), 1 | 0.454, 0.214 (0.46 ; 0.58), 4 | 0.756, 0.197 (0.57 ; 0.95), 2 | 0.709, 0.241 (0.42 ; 0.91), 5 | 0.368, 0.812 (-0.07 ; 1), 4 | 0.580, 0.320 (0.31 ; 0.88), 46 |
| Hemiplegia/paraplegia | 0.670, 0.000 (0.67 ; 0.67), 1 | 0.300, 0.000 (0.30 ; 0.30), 1 | 0.210, 0.258 (0.07 ; 0.54), 3 | 0.1, 0.000 (0.10 ; 0.10), 1 | -0.059, 0.727 (-0.35 ; 0.45), 2 | -0.059, 0.727 (-0.35 ; 0.45), 2 | 0.140, 0.000 (0.14 ; 0.14), 1 | 0.155, 0.370 (-0.03 ; 0.34), 7 | 0.146, 0.352 (-0.07 ; 0.34), 16 |
| Dementia | 0.020, 0.000 (0.02 ; 0.02), 1 | -0.006, 0.371 (-0.24 ; -0.10) 3 | . | . | 0.440, 0.000 (0.44 ; 0.44), 1 | . | . | 0.235, 0.347 (-0.02 ; 0.31), 3 | 0.102, 0.304 (-0.10 ; 0.31), 8 |
| Cancer | 0.710, 0.214 (0.56 ; 0.91), 34 | 0.559, 0.357 (0.35 ; 0.84), 24 | 0.367, 0.628 (-0.11 ; 0.79), 18 | 0.652, 0.174 (0.51 ; 0.85), 18 | 0.722, 0.170 (0.61 ; 0.88), 26 | 0.428, 0.691 (-0.11 ; 0.85), 7 | 0.479, 0.235 (0.21 ; 0.70), 15 | 0.568, 0.248 (0.25 ; 0.87), 57 | 0.563, 0.335 (0.34 ; 0.85), 199 |
| None of the above | 0.829, 0.206 (0.78 ; 0.97), 406 | 0.859, 0.194 (0.84 ; 0.97), 423 | 0.843, 0.182 (0.85 ; 0.97), 467 | 0.853, 0.168 (0.84 ; 0.97), 519 | 0.868, 0.196 (0.82 ; 0.97), 467 | 0.864, 0.159 (0.84 ; 0.97), 449 | 0.844, 0.221 (0.79 ; 0.97), 441 | 0.850, 0.258 (0.82 ; 1), 673 | 0.851, 0.203 (0.82 ; 0.97), 3845 |

**Table S4.** Regression analysis of EQ-5D-5L utility differences by age, sex, and country; POPUP study, January–March 2021

|  | **Belgium** | | **Canada** | | **Germany** | | **Italy** | | **Netherlands** | | **Spain** | | **UK** | | **US** | | **All** | |
| --- | --- | --- | --- | --- | --- | --- | --- | --- | --- | --- | --- | --- | --- | --- | --- | --- | --- | --- |
|  | N=1000 | | N=1000 | | N=1000 | | N=1000 | | N=1000 | | N=1000 | | N=1000 | | N=2000 | | N=9000 | |
|  | **Estimate** | **P-value** | **Estimate** | **P-value** | **Estimate** | **P-value** | **Estimate** | **P-value** | **Estimate** | **P-value** | **Estimate** | **P-value** | **Estimate** | **P-value** | **Estimate** | **P-value** | **Estimate** | **P-value** |
| **Intercept** | 0.849 |  | 0.629 |  | 0.854 |  | 0.896 |  | 0.905 |  | 0.884 |  | 0.654 |  | 0.870 |  | 0.853 |  |
| **Sex** |  |  |  |  |  |  |  |  |  |  |  |  |  |  |  |  |  |  |
| Female | -0.028 | <.0001 | 0.146 | 0.238 | 0.006 | 0.829 | -0.092 | <.0001 | -0.090 | <.0001 | 0.035 | 0.965 | 0.127 | 0.893 | 0.058 | 0.478 | -0.036 | 0.002 |
| Male | ref |  | ref |  | ref |  | ref |  | ref |  | ref |  | ref |  | ref |  | ref |  |
| **Age category** |  |  |  |  |  |  |  |  |  |  |  |  |  |  |  |  |  |  |
| 18-24 | 0.098 | 0.006 | 0.182 | 0.005 | 0.058 | <.0001 | -0.009 | <.0001 | -0.026 | 0.915 | 0.025 | 0.000 | 0.197 | 0.308 | 0.059 | <.0001 | 0.039 | <.0001 |
| 25-34 | 0.045 |  | 0.209 |  | 0.071 |  | -0.003 |  | -0.045 |  | 0.006 |  | 0.114 |  | -0.035 |  | 0.004 |  |
| 35-44 | 0.024 |  | 0.224 |  | 0.023 |  | 0.046 |  | 0.000 |  | 0.003 |  | 0.134 |  | -0.153 |  | -0.025 |  |
| 45-54 | 0.009 |  | 0.195 |  | 0.003 |  | 0.036 |  | -0.038 |  | -0.035 |  | 0.138 |  | 0.016 |  | 0.007 |  |
| 55-64 | 0.036 |  | 0.196 |  | -0.054 |  | 0.035 |  | -0.044 |  | -0.037 |  | 0.100 |  | -0.063 |  | -0.028 |  |
| 65-74 | 0.010 |  | 0.194 |  | ref |  | 0.003 |  | -0.053 |  | ref |  | 0.124 |  | 0.006 |  | 0.007 |  |
| 75+ | ref |  | ref |  | - |  | ref |  | ref |  | - |  | ref |  | ref |  | 0.000 |  |
| **Sex*Age category** |  |  |  |  |  |  |  |  |  |  |  |  |  |  |  |  |  |  |
| Female * 18-24 | -0.066 | 0.255 | -0.141 | 0.563 | 0.050 | 0.457 | 0.086 | 0.002 | 0.006 | 0.512 | -0.049 | 0.896 | -0.282 | 0.042 | -0.068 | <.0001 | -0.004 | <.0001 |
| Female * 25-34 | -0.032 |  | -0.133 |  | 0.020 |  | 0.105 |  | 0.061 |  | -0.037 |  | -0.107 |  | -0.097 |  | 0.014 |  |
| Female * 35-44 | 0.022 |  | -0.132 |  | 0.000 |  | 0.079 |  | -0.013 |  | -0.037 |  | -0.092 |  | 0.095 |  | 0.076 |  |
| Female * 45-54 | -0.049 |  | -0.150 |  | 0.013 |  | 0.052 |  | 0.030 |  | -0.062 |  | -0.135 |  | -0.101 |  | 0.003 |  |
| Female * 55-64 | -0.020 |  | -0.178 |  | -0.031 |  | -0.018 |  | 0.012 |  | -0.031 |  | -0.144 |  | -0.049 |  | 0.026 |  |
| Female * 65-74 | 0.016 |  | -0.157 |  | ref |  | 0.054 |  | 0.066 |  | ref |  | -0.108 |  | -0.089 |  | 0.015 |  |
| Female * 75+ | ref |  | ref |  | - |  | ref |  | ref |  | - |  | ref |  | ref |  | ref |  |

**Figure S1.** Distribution of EQ-5D-5L utility values and EQ VAS scores by country; POPUP study, January–March 2021

Histograms showing the distribution of utility values are presented on the right, while boxplots presenting the distribution of EQ VAS scores are on the left. In the boxplots, dashed lines indicate the mean, solid black lines represent the median, Q1 values are labelled at the bottom right, and Q3 values at the top right of each box.

| **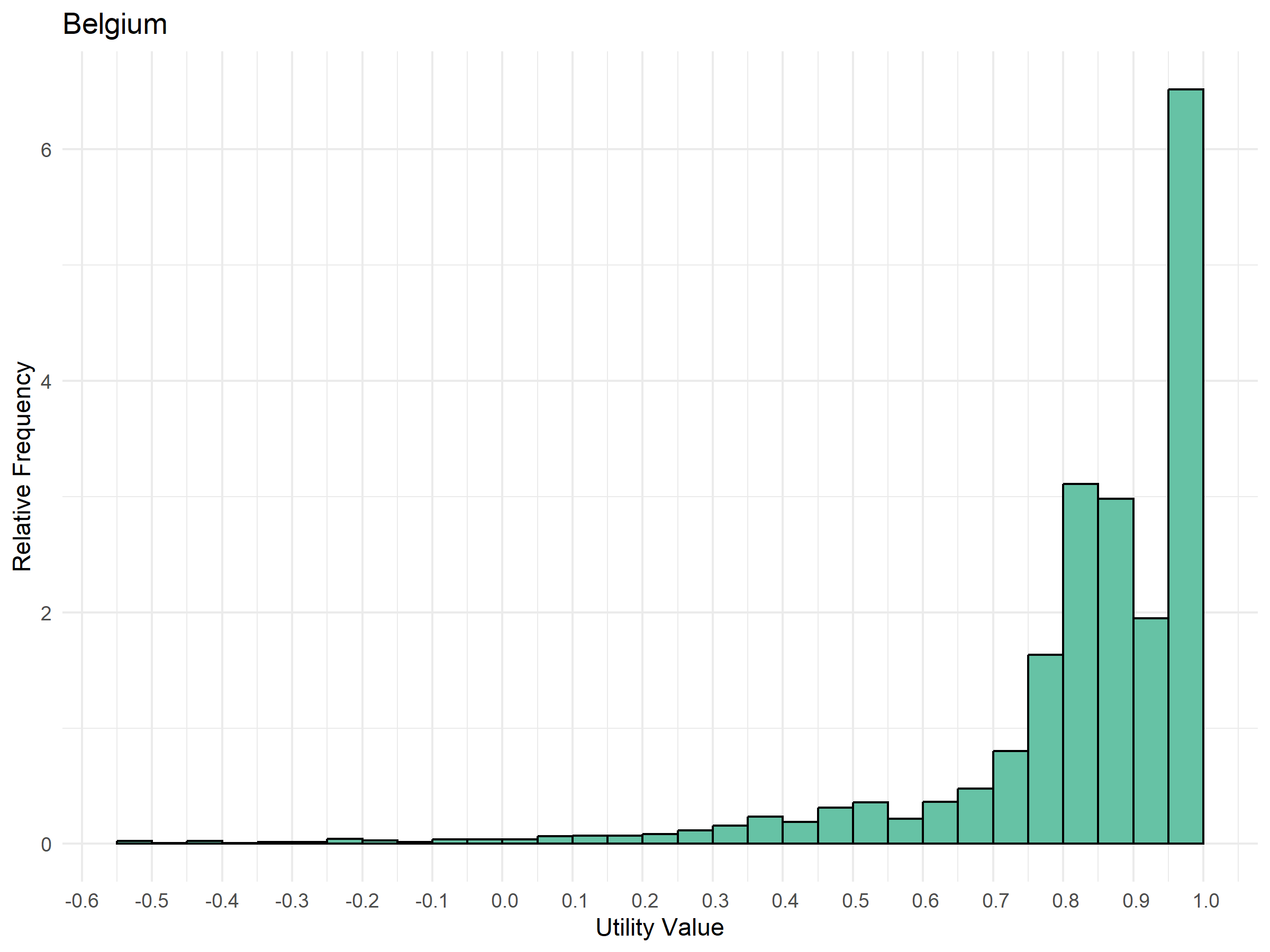** | **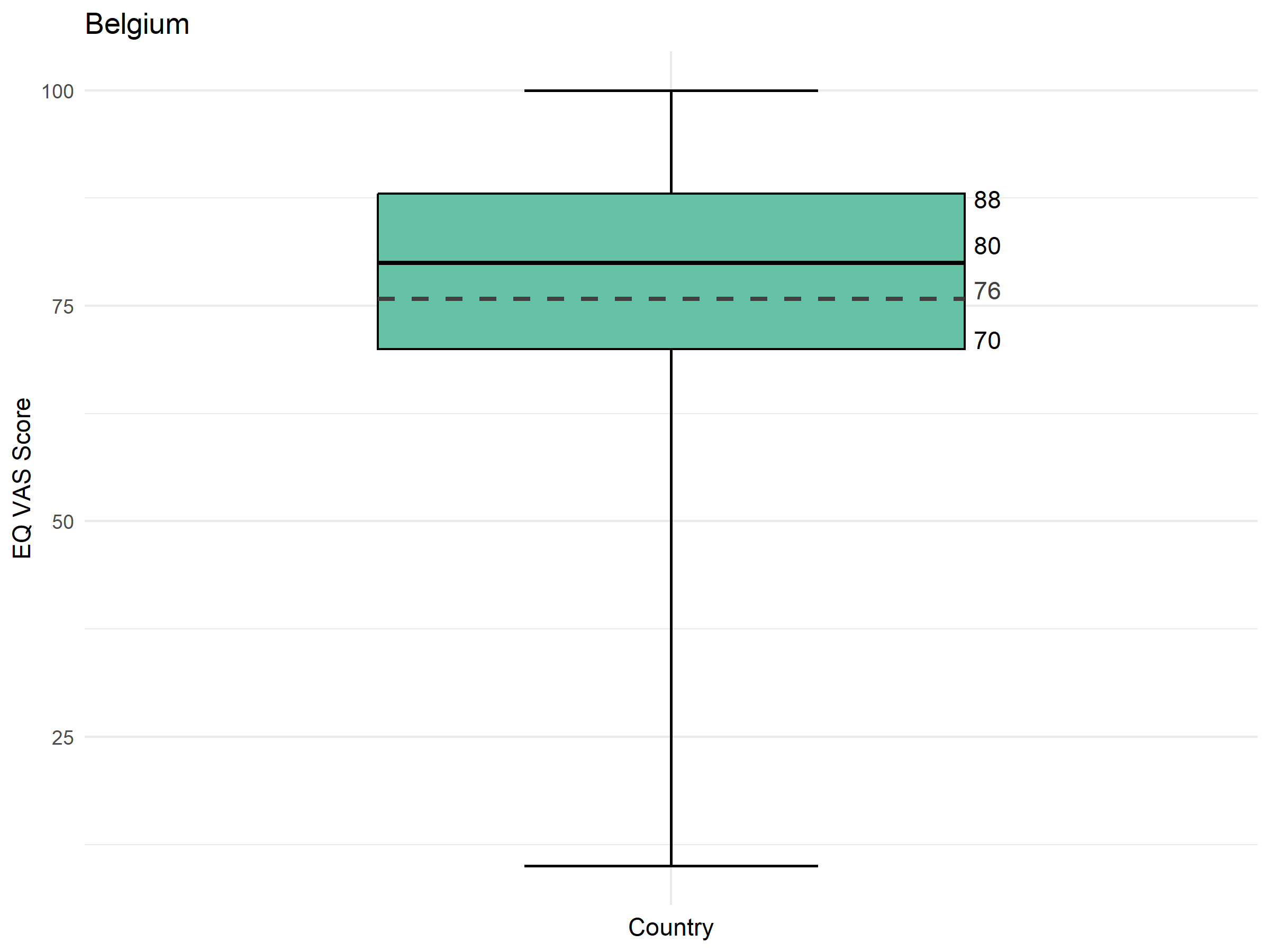** |
| --- | --- |
| **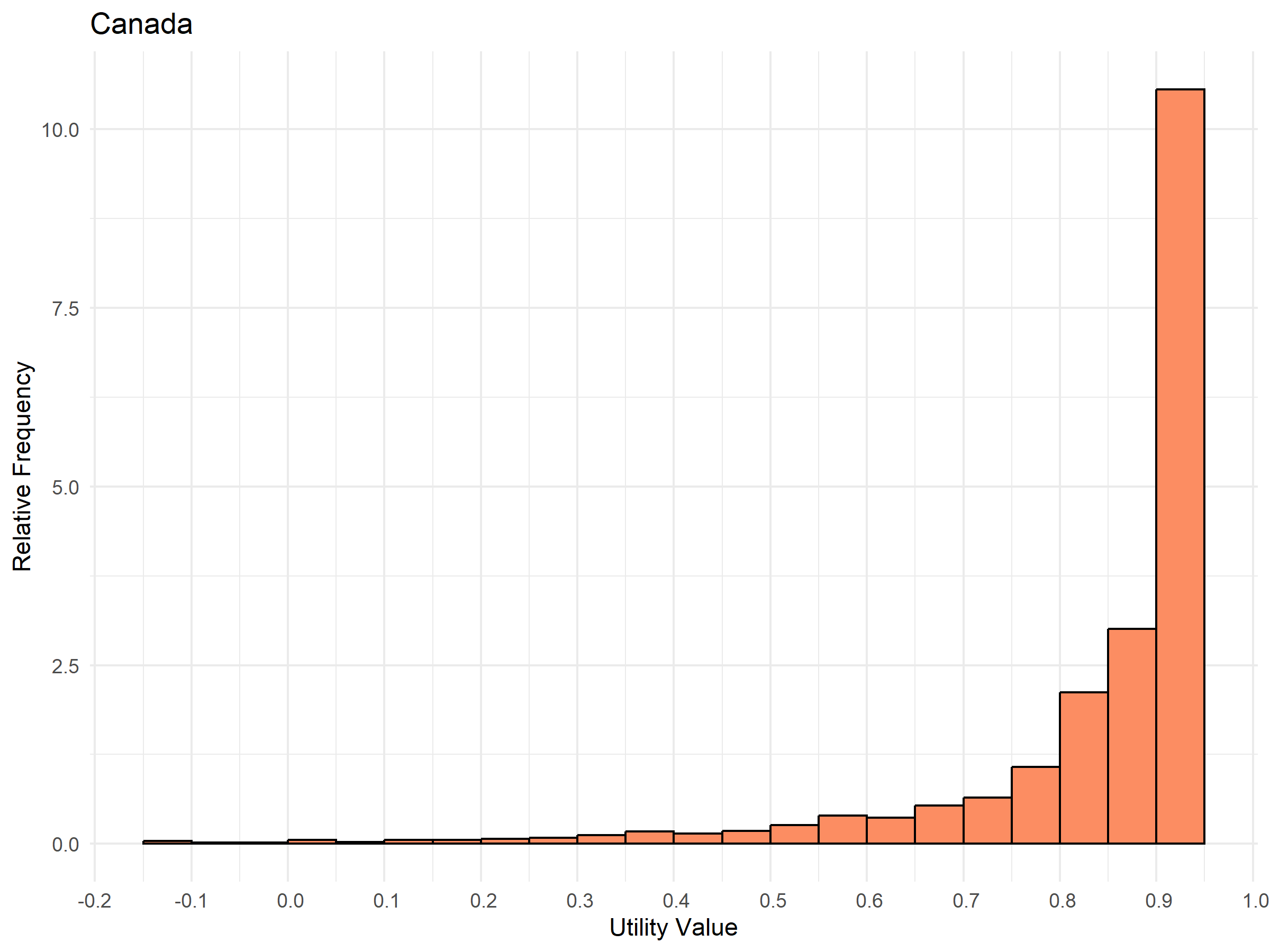** | **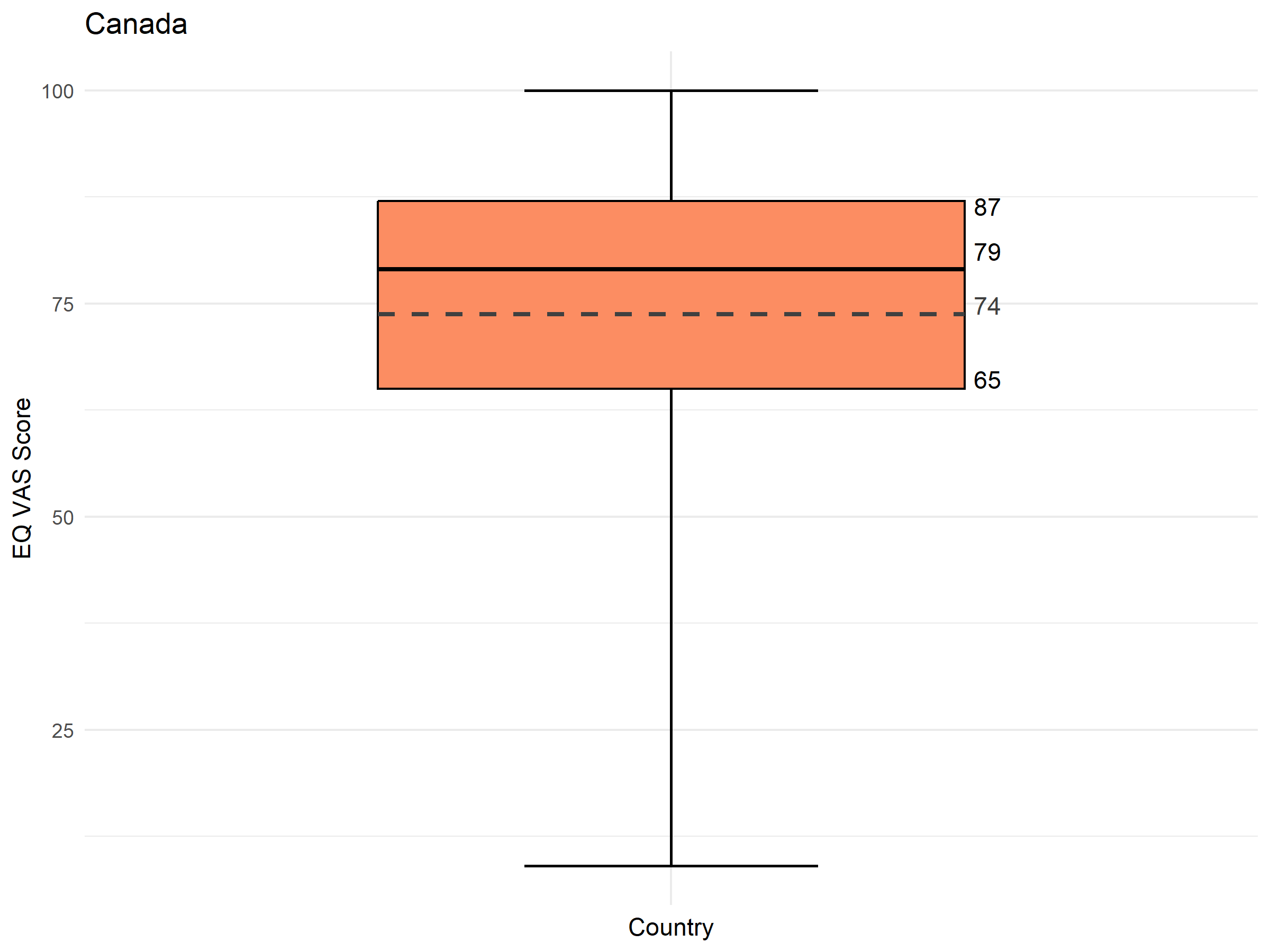** |
| **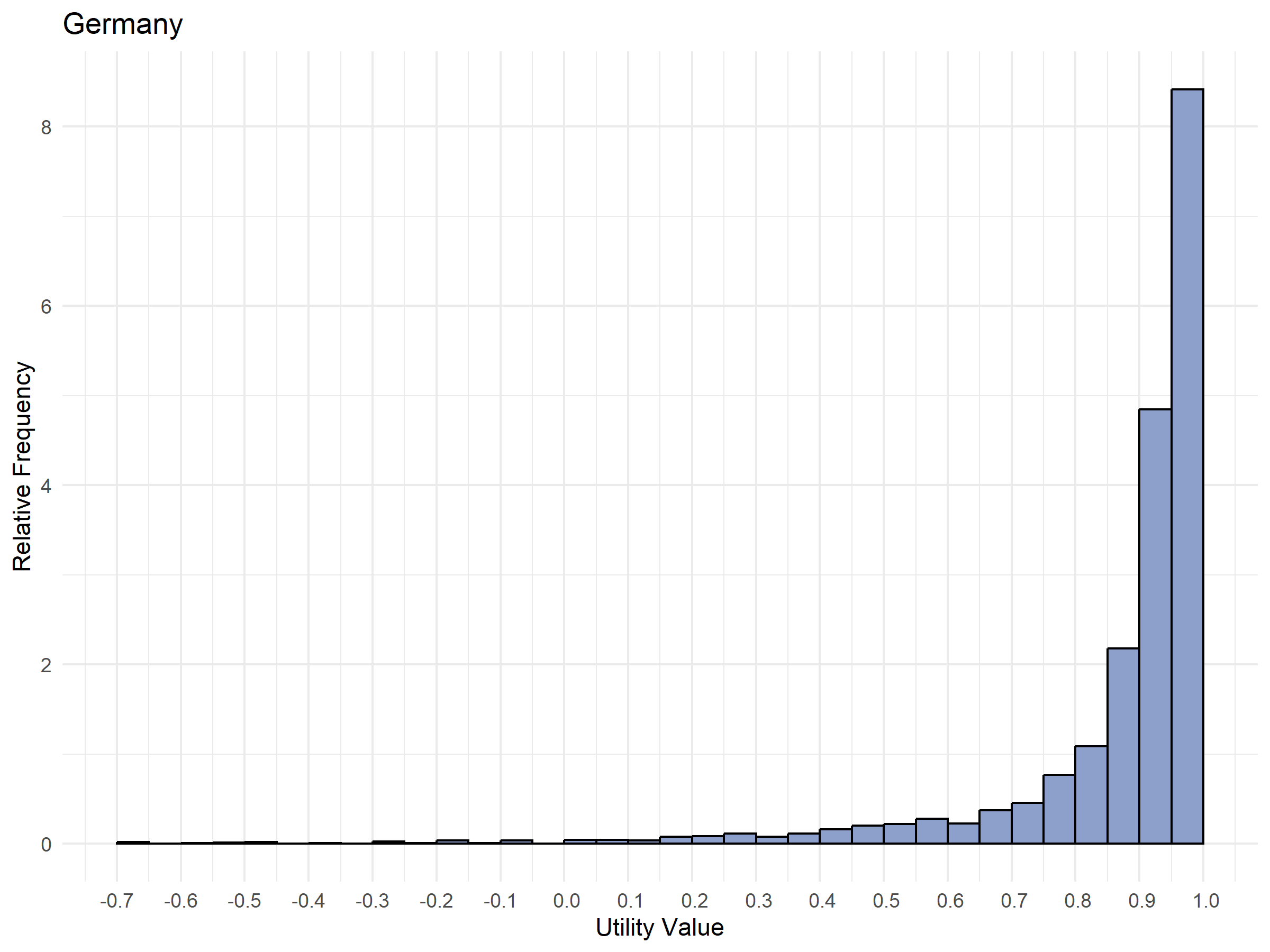** | **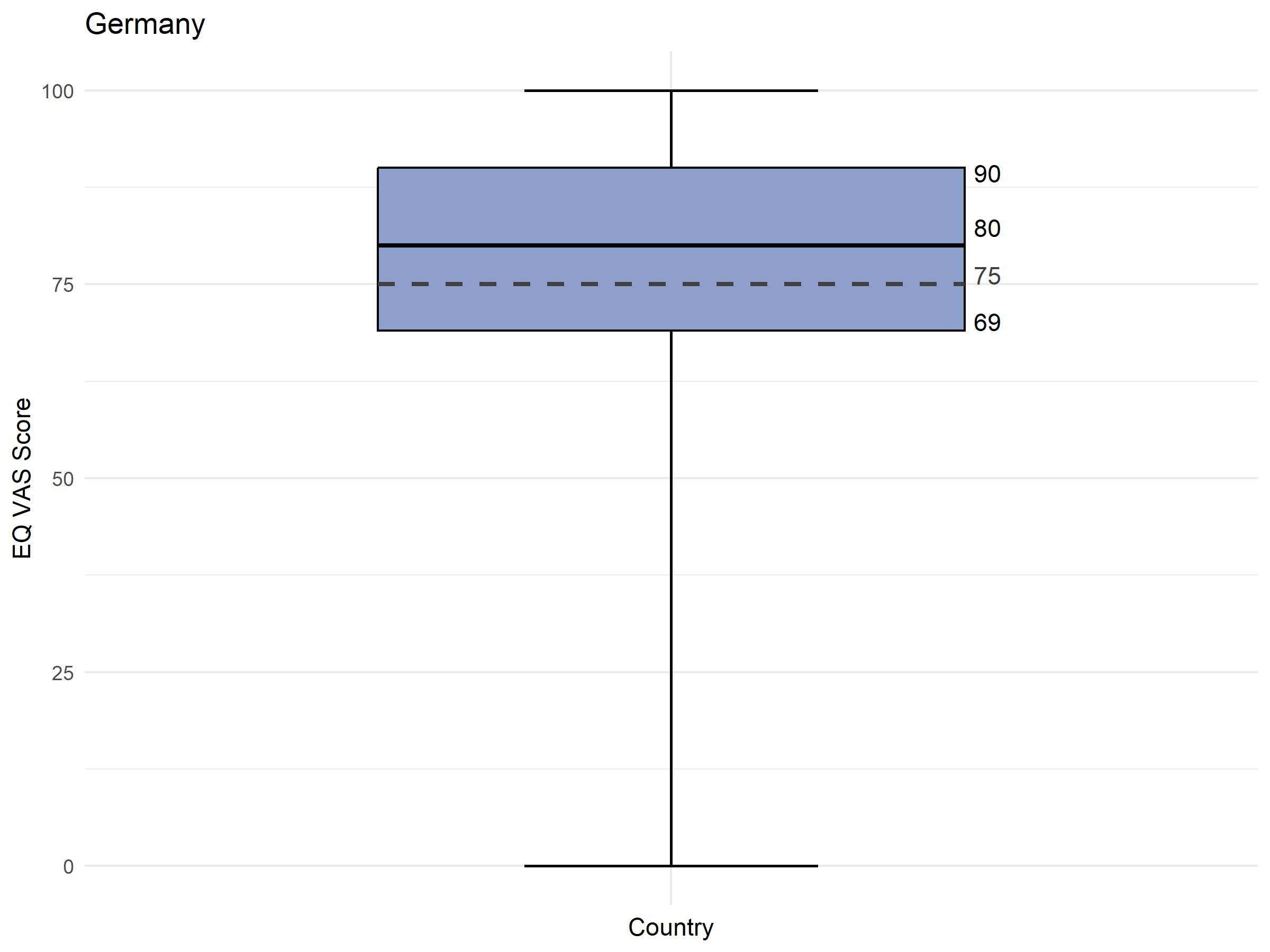** |
| **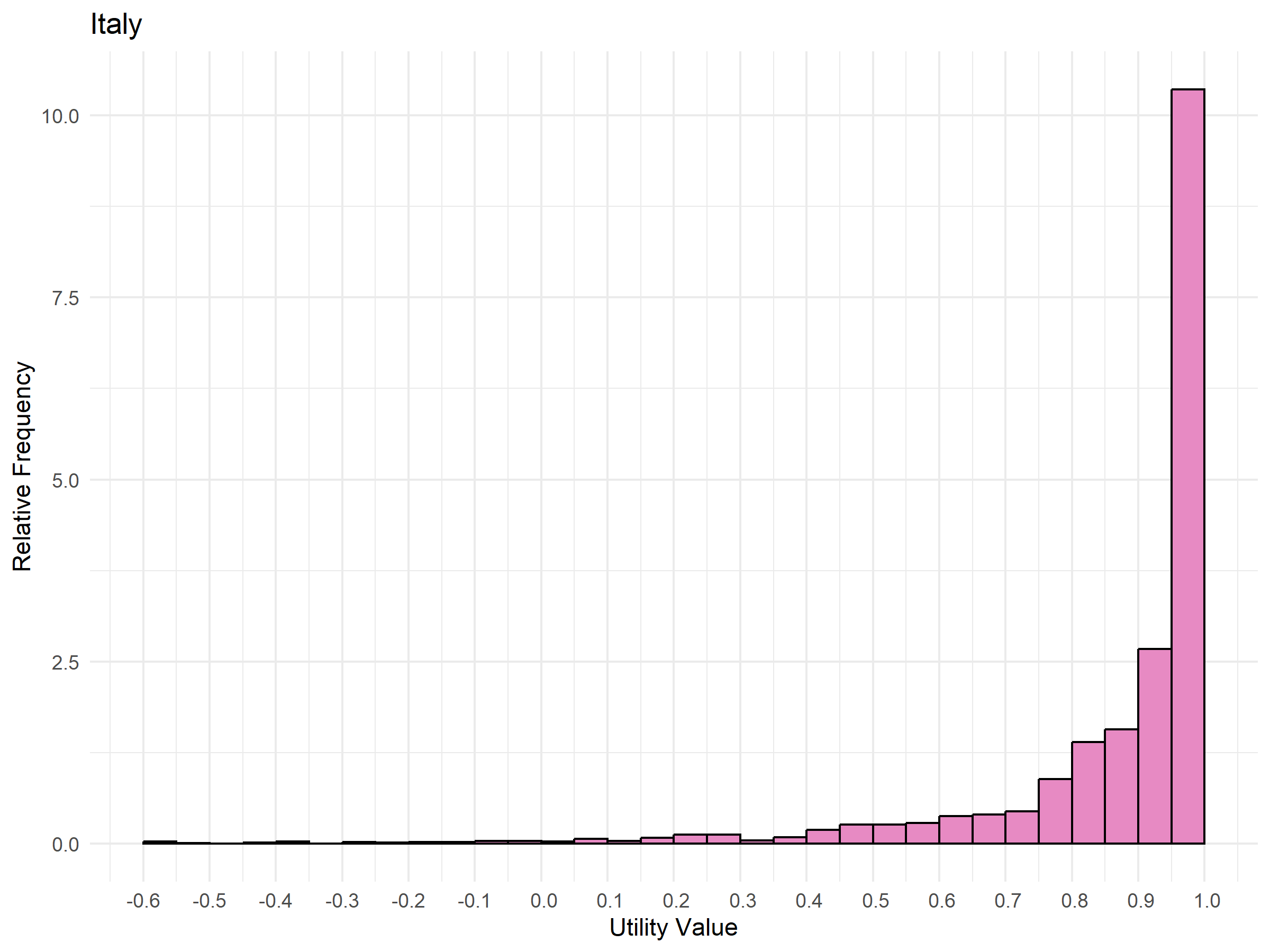** | **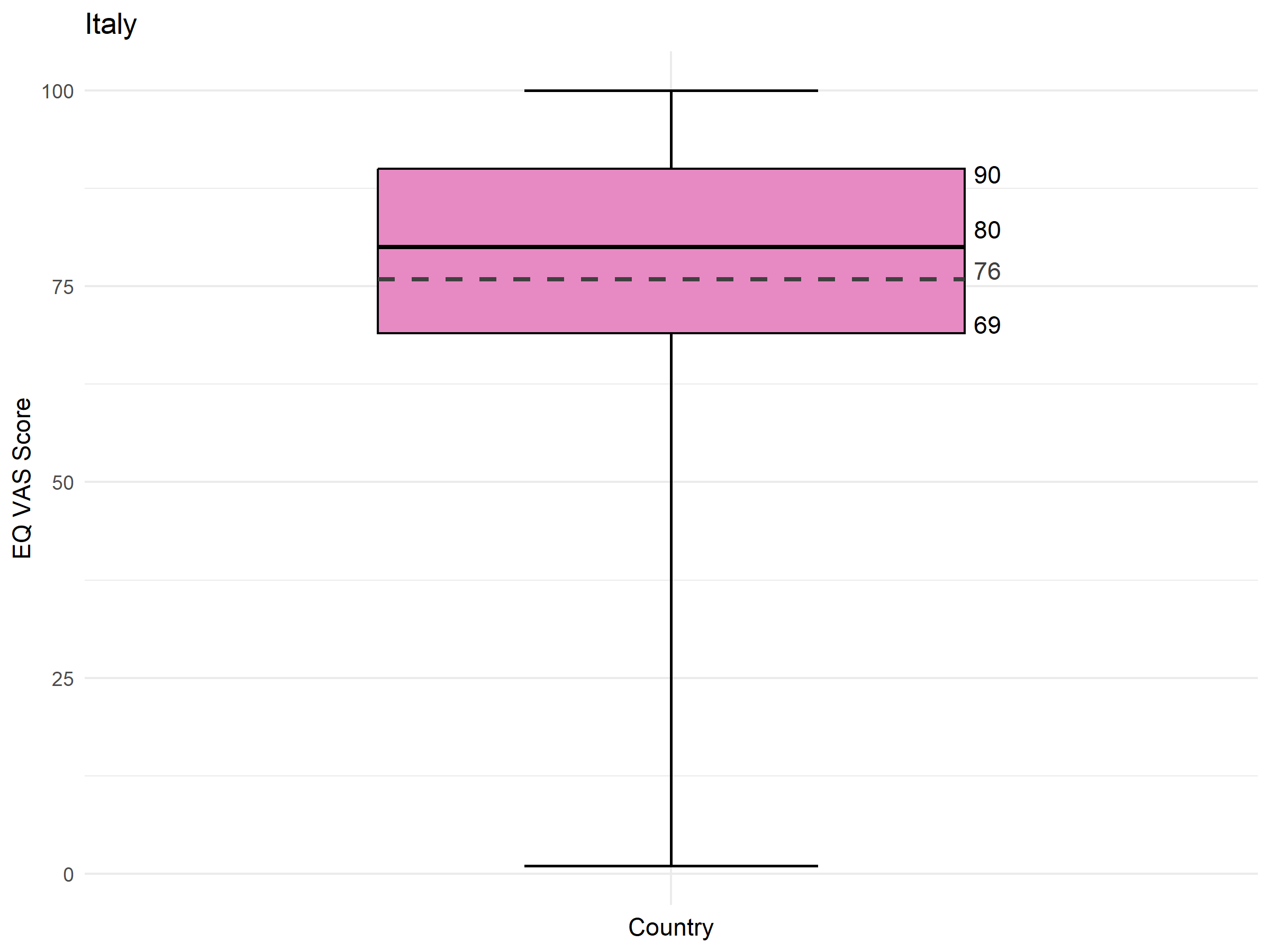** |
| **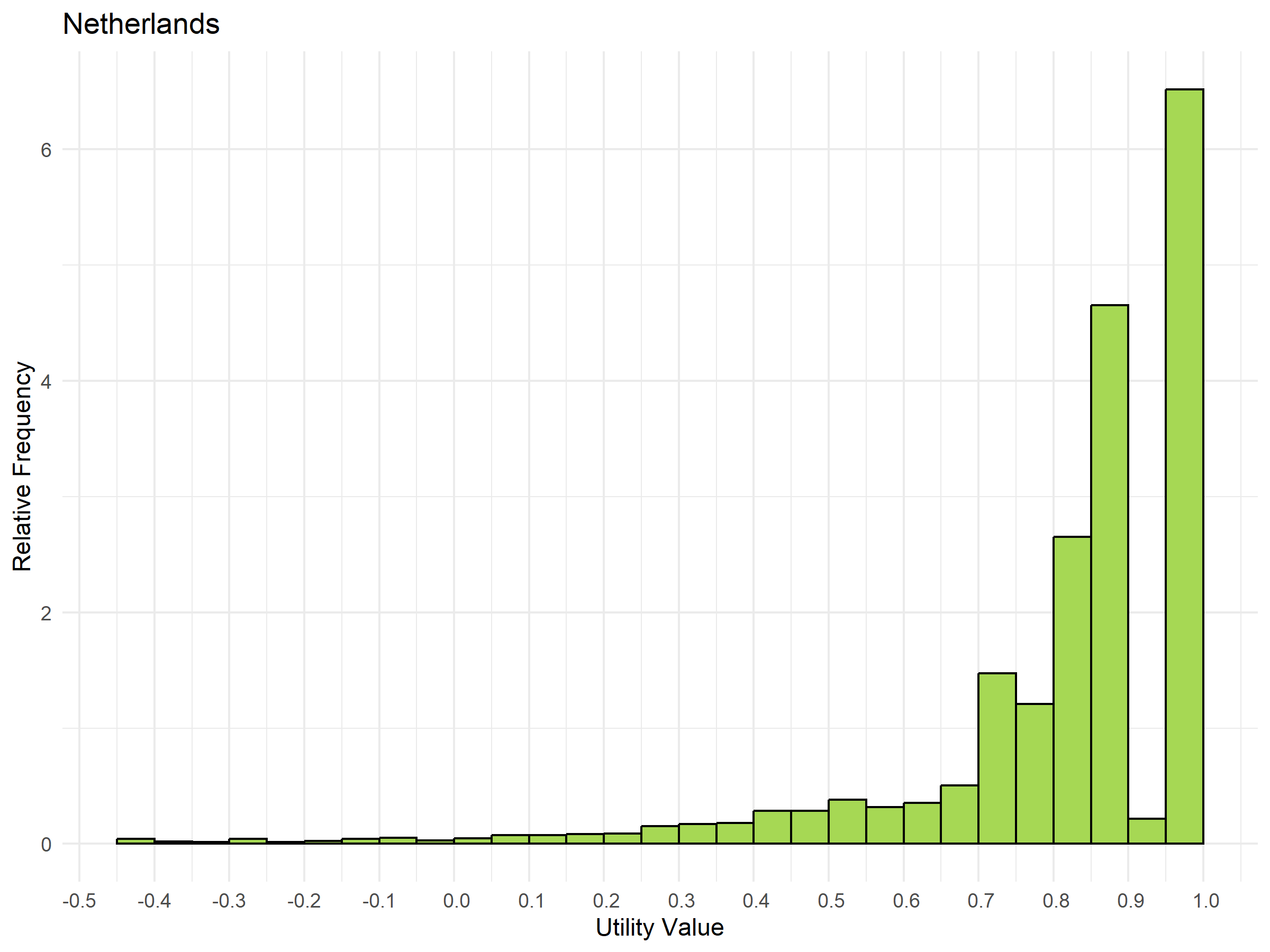** | **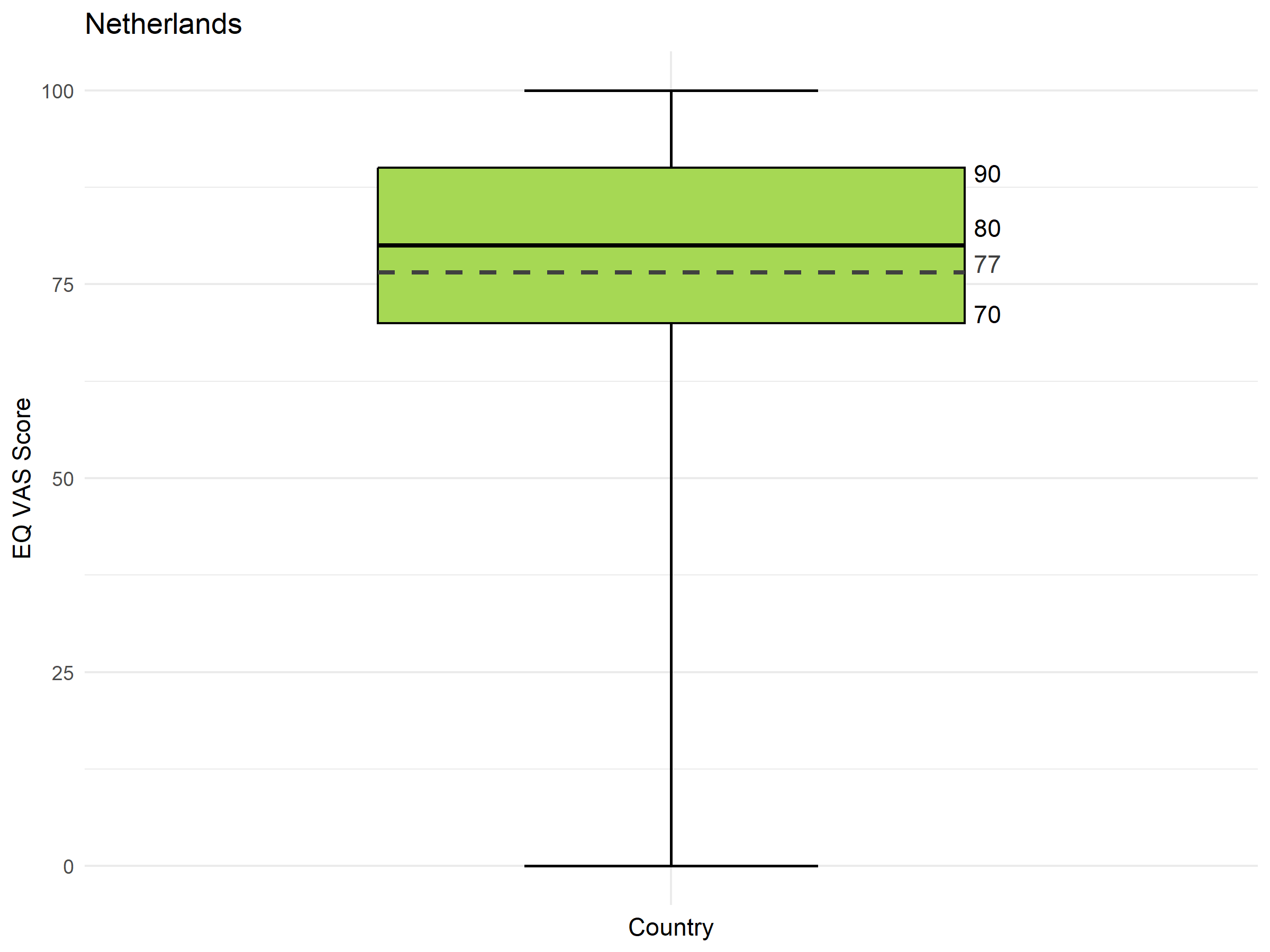** |
| **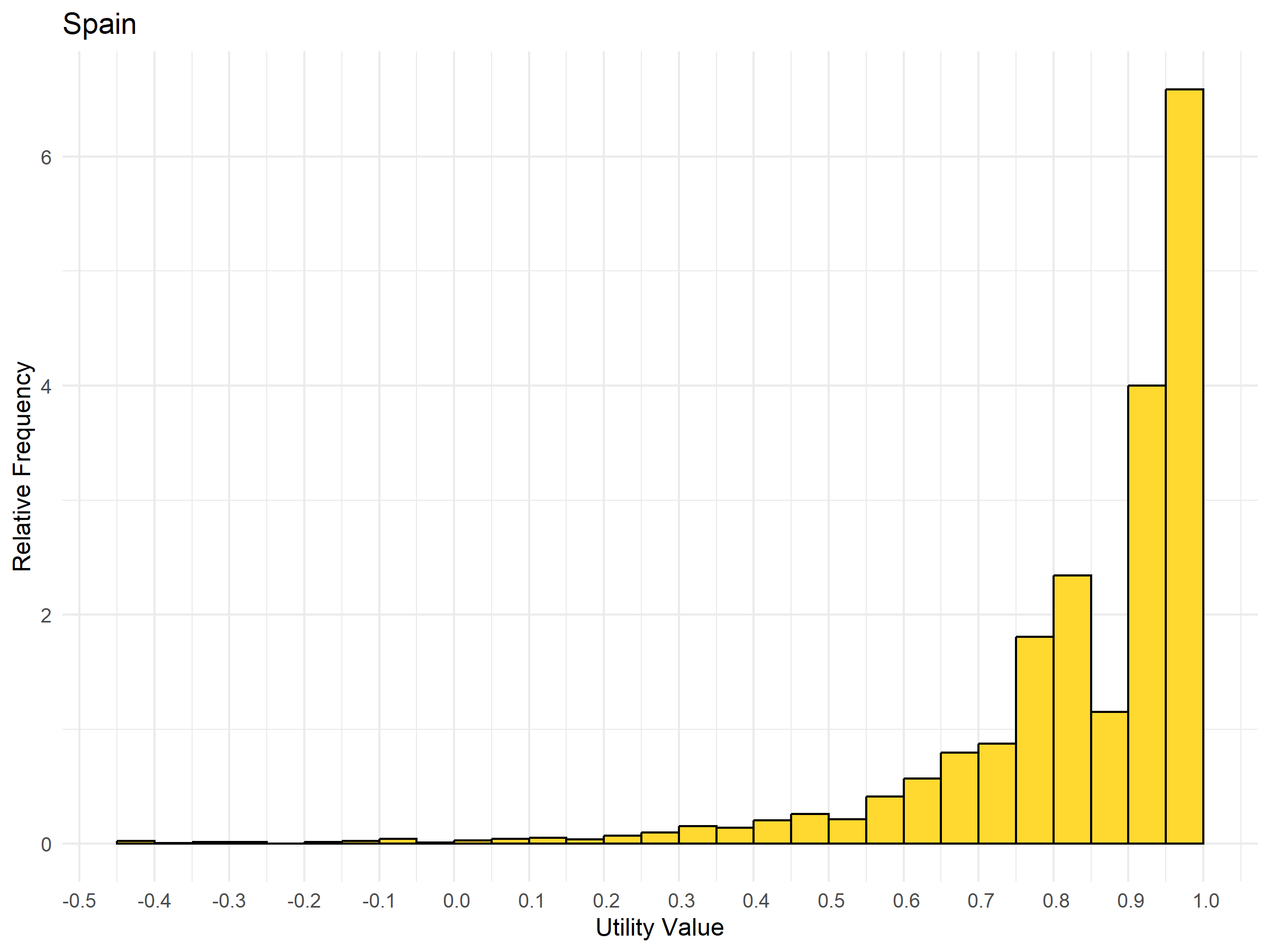** | **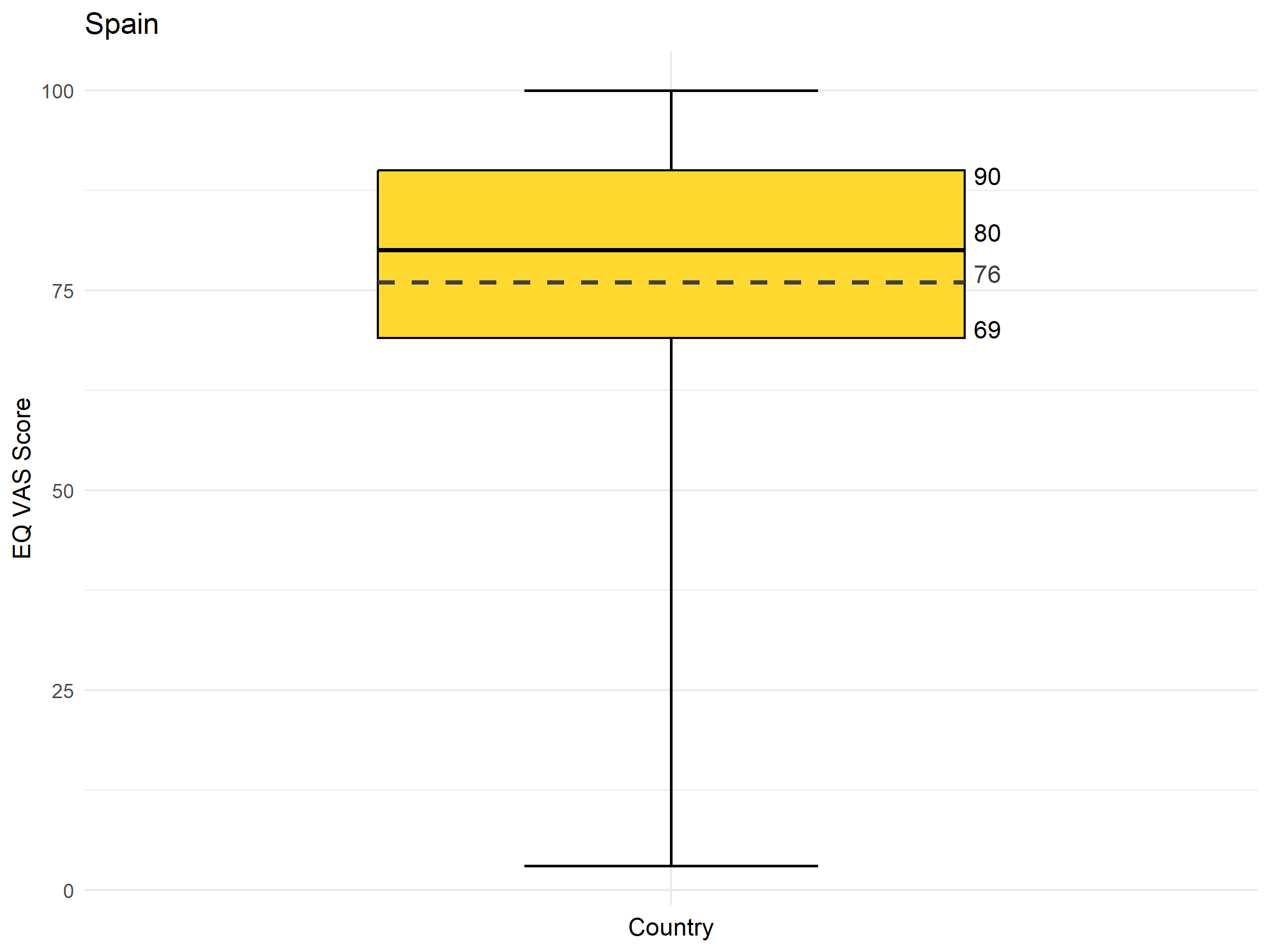** |
| **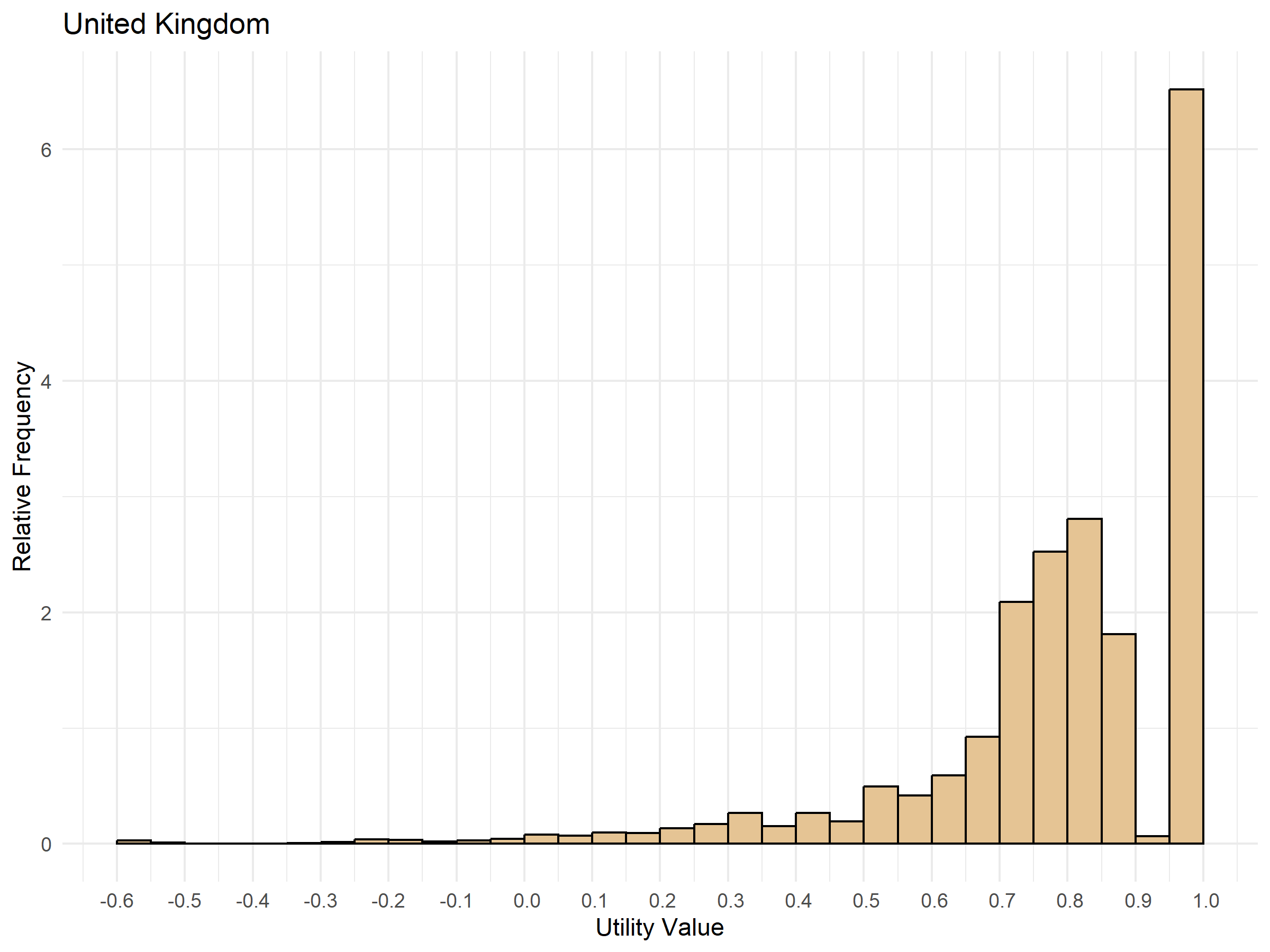** | **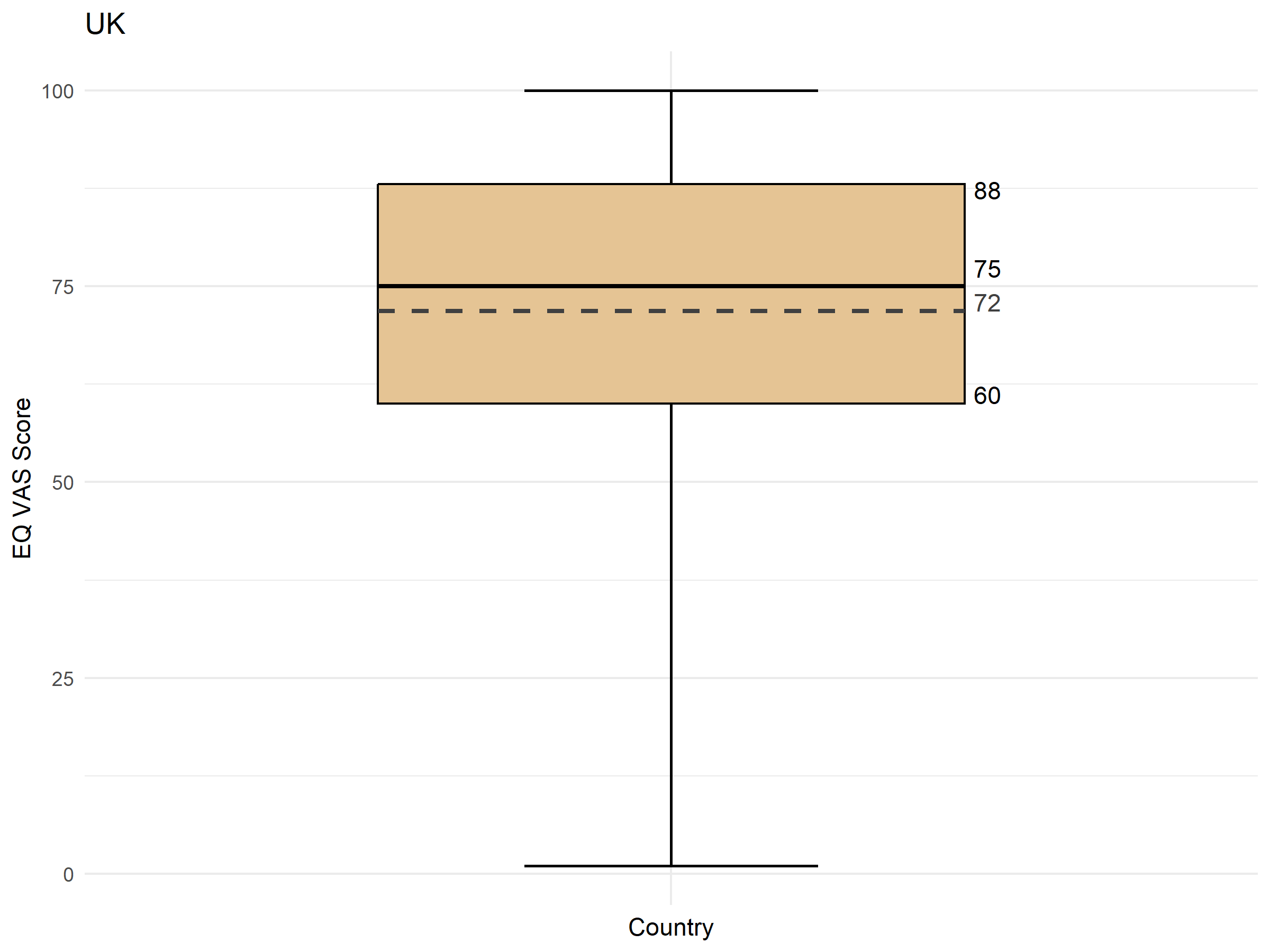** |
| **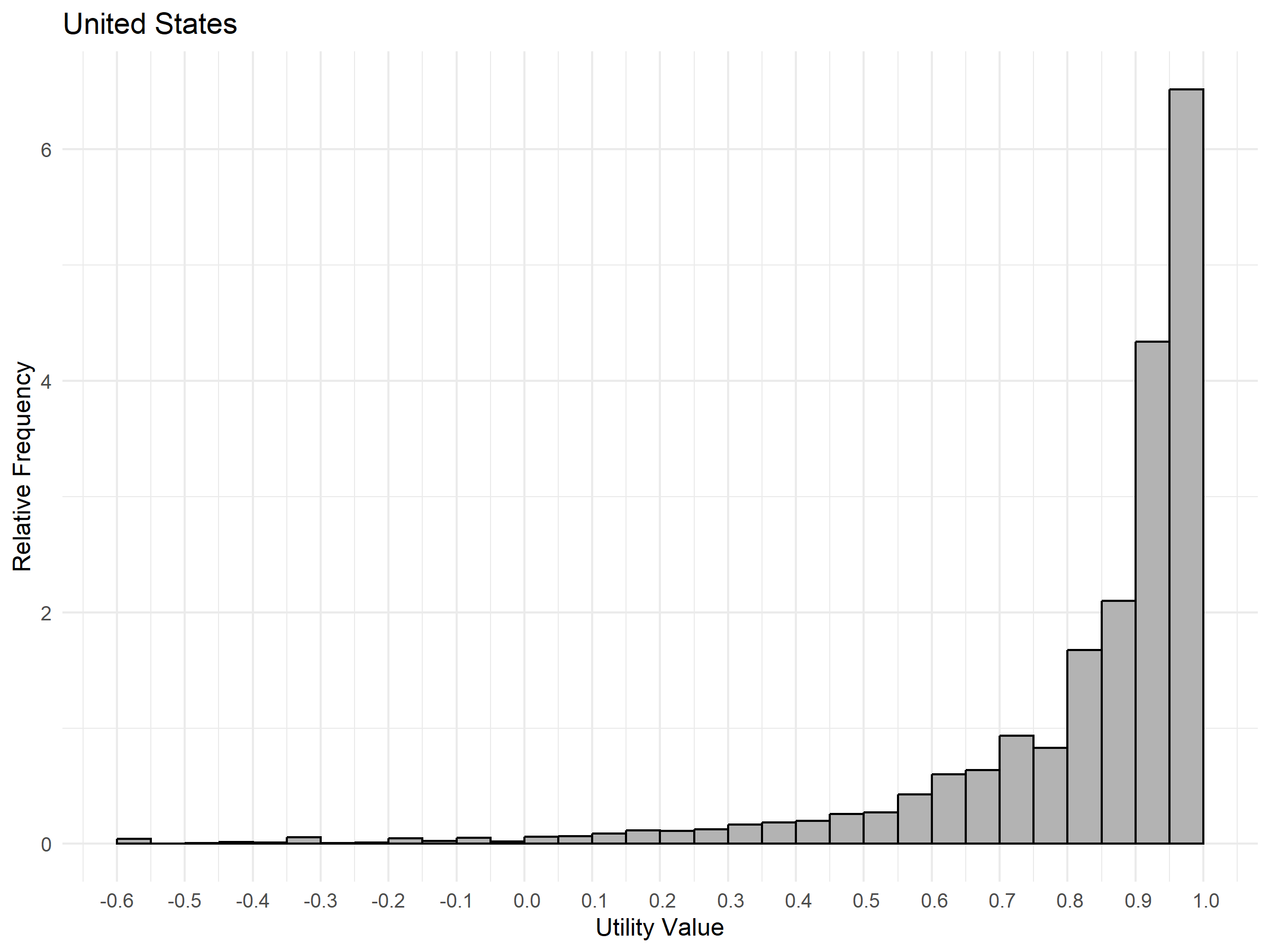** | **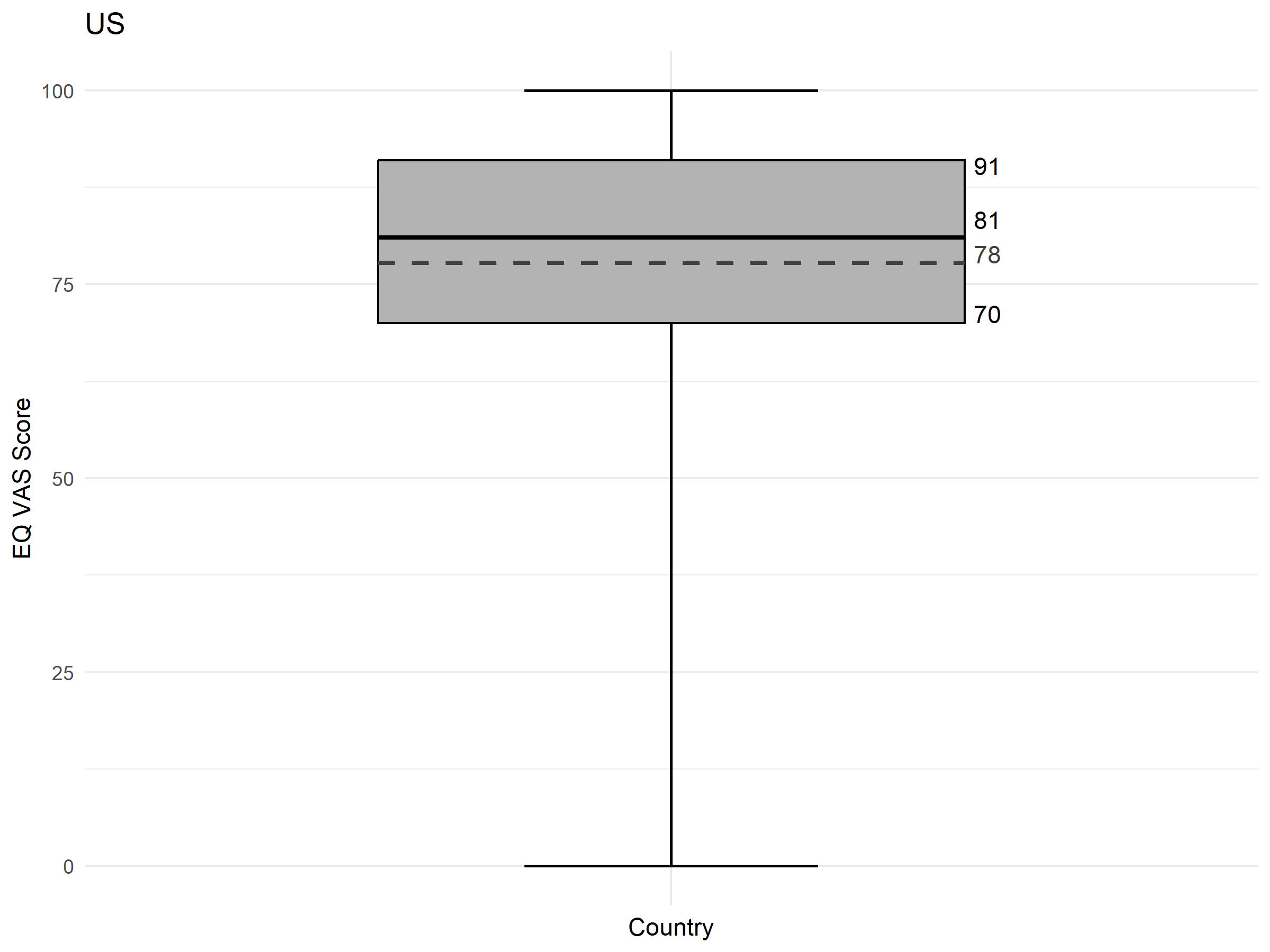** |
